# Gut microbiome features and metabolites in non-alcoholic fatty liver disease among community-dwelling middle-aged and older adults

**DOI:** 10.1186/s12916-024-03317-y

**Published:** 2024-03-07

**Authors:** Fangfang Zeng, Xin Su, Xinxiu Liang, Minqi Liao, Haili Zhong, Jinjian Xu, Wanglong Gou, Xiangzhou Zhang, Luqi Shen, Ju-Sheng Zheng, Yu-ming Chen

**Affiliations:** 1 Department of Public Health and Preventive Medicine, School of Medicine, Jinan University, No.601 Huangpu Road West, Guangzhou, 510632 China; 2 Department of Epidemiology, Guangdong Provincial Key Laboratory of Food, Nutrition and Health, School of Public Health, Sun Yat-Sen University, Guangzhou, 510275 China; 3 Zhejiang Key Laboratory of Multi-Omics in Infection and Immunity, School of Medicine and School of Life Sciences, Westlake University, Hangzhou, 310030 China; 4 Institute of Epidemiology, Helmholtz Zentrum München-German Research Center for Environmental Health, Ingolstädter Landstr. 1, 85764 Neuherberg, Germany; 5 Big Data Decision Institute, Jinan University, No.601 Huangpu Road West, Guangzhou, 510632 China

**Keywords:** Non-alcoholic fatty liver disease, Gut microbiota feature, Gut metabolites, Machine learning algorithms, 16S rRNA gene sequence

## Abstract

**Background:**

The specific microbiota and associated metabolites linked to non-alcoholic fatty liver disease (NAFLD) are still controversial. Thus, we aimed to understand how the core gut microbiota and metabolites impact NAFLD.

**Methods:**

The data for the discovery cohort were collected from the Guangzhou Nutrition and Health Study (GNHS) follow-up conducted between 2014 and 2018. We collected 272 metadata points from 1546 individuals. The metadata were input into four interpretable machine learning models to identify important gut microbiota associated with NAFLD. These models were subsequently applied to two validation cohorts [the internal validation cohort (*n* = 377), and the prospective validation cohort (*n* = 749)] to assess generalizability. We constructed an individual microbiome risk score (MRS) based on the identified gut microbiota and conducted animal faecal microbiome transplantation experiment using faecal samples from individuals with different levels of MRS to determine the relationship between MRS and NAFLD. Additionally, we conducted targeted metabolomic sequencing of faecal samples to analyse potential metabolites.

**Results:**

Among the four machine learning models used, the lightGBM algorithm achieved the best performance. A total of 12 taxa-related features of the microbiota were selected by the lightGBM algorithm and further used to calculate the MRS. Increased MRS was positively associated with the presence of NAFLD, with odds ratio (OR) of 1.86 (1.72, 2.02) per 1-unit increase in MRS. An elevated abundance of the faecal microbiota (*f__veillonellaceae*) was associated with increased NAFLD risk, whereas *f__rikenellaceae*, *f__barnesiellaceae*, and *s__adolescentis* were associated with a decreased presence of NAFLD. Higher levels of specific gut microbiota-derived metabolites of bile acids (taurocholic acid) might be positively associated with both a higher MRS and NAFLD risk. FMT in mice further confirmed a causal association between a higher MRS and the development of NAFLD.

**Conclusions:**

We confirmed that an alteration in the composition of the core gut microbiota might be biologically relevant to NAFLD development. Our work demonstrated the role of the microbiota in the development of NAFLD.

**Supplementary Information:**

The online version contains supplementary material available at 10.1186/s12916-024-03317-y.

## Background

Non-alcoholic fatty liver disease (NAFLD) is the most common chronic liver disease affecting 25% of general adults worldwide and is one of the major risk factors for various diseases, including cardiovascular diseases (CVDs) and liver cancers, resulting in an increasing number of global mortalities [1]. In addition to genetic predisposition and diet, the gut microbiota has emerged as one of the environmental factors contributing to the development of NAFLD [2]. Currently, there is no approved therapy; however, manipulating the gut microbiota in conjunction with lifestyle factors may present an alternative intervention for NAFLD.

The potential causal effect of the gut microbiota composition on the development of NAFLD was first revealed and supported by evidence from animal faecal microbiota transplantation experiments that induced hepatic macrovesicular steatosis in mice [3] and a population study that revealed a significant increase in the abundance of alcohol-producing bacteria in non-alcoholic steatohepatitis (NASH) patient microbiomes, along with elevated blood ethanol concentrations [4]. Subsequently, numerous animal studies and a handful of human studies suggest the beneficial role of probiotics, prebiotics, or synbiotics in reshaping the gut microbiota composition and activities, thus improving the liver phenotype [5]. Several mechanisms have been proposed to explain the role of the gut microbiota in NAFLD development, including affecting the amount of energy absorbed from the diet, altering intestinal permeability to lead to bacterial migration and the parallel release of toxic bacterial products, changing the expression of genes involved in de novo lipogenesis and metabolic signalling pathways, producing ethanol in the intestine, and interacting with innate immunity [5, 6].

In addition, the various metabolites produced by the gut microbiota may modulate NAFLD susceptibility, for example, fermentation of indigestible carbohydrates (e.g. dietary fibre) by gut microbiota yields metabolites such as short-chain fatty acids (SCFAs) [7], propionate, butyrate, and succinate [8], which might have beneficial roles in body weight control, inflammatory status, glucose, and lipid homeostasis [9]. The dysregulation of bile acid metabolism in NAFLD may lead to increased energy expenditure and a chronic inflammatory state [10], while elevated production of deoxycholic acid in NASH patients, attributed to the enrichment abundance of bacteria producing secondary bile acids, inhibited FXR signalling, impeding lipid and glucose metabolism in the liver and intestine [11]. Dysregulation of amino acids and choline leads to lipid accumulation and chronic inflammation [10].

Researchers worldwide have made great efforts to investigate what makes a ‘good’ gut microbiome in human [12], as has been done for NAFLD. Many epidemiological studies have assessed the distribution of the gut microbiota between healthy individuals and NAFLD patients. Reduced bacterial α- or β-diversity in NAFLD patients has been observed in some, but not all studies [13–15]. For specific microbial taxa, a meta-analysis of 54 studies (8894 participants) revealed the depletion of anti-inflammatory microbes (i.e. *Ruminococcaceae* and *Coprococcus*) and the enrichment of proinflammatory microbes (i.e. *Fusobacterium* and *Escherichia*) in patients with NAFLD [16]; however, there was significant interstudy heterogeneity, and most of the previous evidence was based on cross-sectional studies with limited sample sizes.

Another limitation of most of the previous studies is the application of traditional statistical approaches which typically consider the effects of each bacterial population separately but do not adequately account for the interactions among the microbiota, even interactions with multiple lifestyle factors, complex laboratories, and clinical parameters [17]. The development of interpretable machine learning (ML) algorithms based on bacterial abundance has made significant progress [18], and these algorithms have emerged as useful tools for identifying gut microbiome features and aided in the diagnosis of specific diseases, such as type 2 diabetes (T2DM) [19] and cancer [17]. A nested case–control study with 90 pairs of matched participants with/without NAFLD progression provided evidence of the association between NAFLD progression and gut microbiota features identified by random forest (RF) [20]. However, additional systematic comparisons of ML algorithms are needed to explore the association between NAFLD development and faecal microbiota signatures.

This study aimed to identify the human gut microbiota features associated with NAFLD based on different machine learning models (RF, support vector machine (SVM), logistic regression, and lightGBM models). We also examined whether the selected features in the model were biologically relevant to faecal or serum metabolites. In addition, an animal model involving faecal microbiota transplantation (FMT) was used to verify the causal effect of gut microbiota from NAFLD patients on the liver phenotype in high-fat diet-induced NAFLD mice.

## Methods

### Study design and oversight

**Fig. 1:**
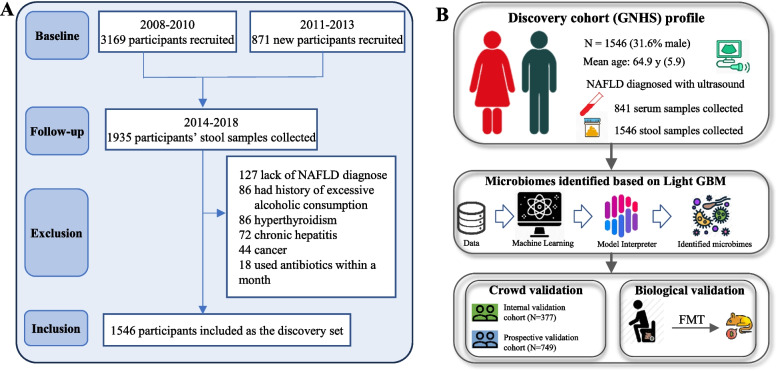
Flow chart. The flow chart in **A** shows the screening process used for the discovery cohort (Guangzhou Nutrition and Health Study), and **B** illustrates the study design

The present study, outlined as a flow chart in Fig. 1, can be divided into the following steps: participant selection, data collection, ML algorithm construction, feature identification, correlation and logistic regression analyses, metabolomic analysis, and mouse validation. We constructed several distinct cohorts, including discovery, internal validation (NAFLD diagnosed with magnetic resonance imaging [MRI]), and prospective validation cohorts for the development and validation of the method.

### Study participants

The discovery set was built based on the Guangzhou Nutrition and Health Study (GNHS). A total of 4048 participants aged 40–80 years were recruited from multiple communities in Guangzhou, China during 2008–2013 through local advertisements, health lectures, and referral methods and followed up every 3 years [21]. All participants were invited by phone to Sun Yat-sen University for face-to-face interviews, specimen collection, and physical examination. For more details about GNHS, please refer to our previous articles [22]. Faecal samples for multiomics assessments were collected during the period of follow-up spanning from 2014 to 2018 (*n* = 1939). A total of 393 participants were excluded because they (i) lacked ultrasound diagnosed NAFLD data (*n* = 127); (ii) had a history of excessive alcohol consumption (≥ 30 g/day for men or ≥ 20 g/day for women) (*n* = 86); (iii) used antibiotics within a month prior to the survey (*n* = 18); and (iv) were suffering from other diseases (e.g. chronic hepatitis [*n* = 72], cancer [*n* = 44], and hyperthyroidism [*n* = 86]). Ultimately, 1546 participants were included in the discovery cohort, 56.1% (867/1546) of the participants were diagnosed with NAFLD by abdominal ultrasonography, whereas those without significant hepatic steatosis based on abdominal ultrasound (US) diagnosis were served as the controls (details are shown in Fig. 1A). The internal validation cohort included 377 participants who underwent MRI at the fourth follow-up (2019–2021) to confirm the NAFLD diagnosis. Moreover, a prospective internal validation cohort consisted of 749 individuals who were without NAFLD at baseline based on the abdominal ultrasound examination were followed for a median of 8.7 years.

The study protocol of the GNHS project was approved by the Ethics Committee of the School of Public Health at Sun Yat-sen University (2,018,048) and registered in the ClinicalTrials.gov database (NCT03179657). All procedures adhered to the principles outlined in the Declaration of Helsinki. Written informed consent was obtained from all participants prior to the start of the investigation.

### Collection and measurement of metadata

The metadata included 5 demographic characteristics, 3 anthropometry factors, 5 blood parameters, 3 lifestyle habits, one physical activity, and 6 dietary indicators (additional details are shown in Table 1). Demographic characteristics and lifestyle habits were investigated via self-administered questionnaires. Participants were first asked whether they consumed alcohol or not, after which the frequency and amount of alcohol consumed were investigated. The average amount of alcohol (g/week) = was calculated as the average alcohol consumption frequency × average amount of alcohol consumed per drinking day [%ABV (alcohol by volume) × 0.79336 (g/mL) × volume (mL)]. Colorimetric methods were used to measure four blood lipids and sugar levels with a Hitachi 7600–010 automated analyser (Hitachi, Tokyo, Japan). Daily physical activity was assessed using the physical activity questionnaires (PAQs), and the metabolic equivalent (MET) intensity was also calculated. Alcohol consumption was self-reported. Daily dietary intake was evaluated using the Food Frequency Questionnaire (FFQ).

**Table 1:** Characteristic of the participants

	**Discovery cohort**	**Internal validation cohort**	**Prospective validation cohort**
No. of participants	1546	377	749
Non-alcoholic fatty liver disease case subjects, *n* (%)	867 (56.1)	126 (33.4)	266 (35.5)
Age (years)	64.9 (5.9)	65.0 (5.1)	64.6 (6.1)
Sex, *n* (%)			
Female	1057(68.4)	277 (73.5)	523 (69.8)
Male	489 (31.6)	100 (26.5)	226 (30.2)
Marital status,* n* (%)			
Married	1326 (85.8)	335 (88.9)	640 (85.4)
others	216 (14.0)	42 (11.1)	109 (14.6)
Education, *n* (%)			
Middle school or less	419 (27.1)	87 (23.1)	208 (27.8)
High school or professional college	706 (45.7)	175 (46.4)	339 (45.2)
University	408 (26.4)	115 (30.5)	202 (27.0)
Income (yuan/month/person), *n* (%)			
≤ 500	323 (20.9)	83 (22.0)	9 (1.2)
501–1500	751 (48.6)	220 (56.4)	144 (19.2)
1501–3000	69 (4.5)	8 (2.1)	337 (45.0)
> 3000	368 (23.8)	62 (16.4)	259 (34.6)
BMI, kg/m^2^	23.5 ± 3.1	23.1 ± 3.0	22.3 ± 2.6
Waist circumference, cm	84.8 ± 8.9	83.9 ± 8.7	81.5 ± 8.3
Hip circumference, cm	91.5 ± 12.1	90.6 ± 7.7	89.9 ± 15.5
Fasting glucose, mmol/L	5.5 ± 1.3	5.5 ± 1.2	5.3 ± 1.2
TC, mmol/L	5.5 ± 1.1	5.6 ± 1.1	5.6 ± 1.1
LDL, mmol/L	3.6 ± 1.0	3.6 ± 1.0	3.7 ± 1.0
TG, mmol/L	1.6 ± 1.1	1.6 ± 1.3	1.4 ± 0.9
HDL, mmol/L	1.6 ± 1.1	1.6 ± 1.3	1.4 ± 0.9
Current smoking status, *n* (%)	110 (7.1)	17 (4.5)	51 (6.8)
Current tea drinking, *n* (%)	833 (53.9)	198 (52.5)	388 (51.8)
Current alcohol drinking, *n* (%)	110 (7.1)	17 (4.5)	51 (6.8)
Physical activity, MET	18.9 (6.9)	19.3 (7.0)	19.6 (7.0)
Total energy intake, kcal/day	1719.3 ± 581.2	1761.7 ± 503.1	1715.9 ± 581.2
Vegetable intake, g/day	332.5 ± 166.7	327.1 ± 160.4	334.1 ± 164.8
Fish intake, g/day	41.4 ± 42.2	44.3 ± 57.5	43.2 ± 51.9
Red and processed meat intake, g/day	80.3 ± 52.7	79.6 ± 43.6	82.9 ± 61.1
Fruit intake, g/day	152.7 ± 105.1	150.0 ± 107.7	151.3 ± 101.0
Yogurt intake, g/day (dry weight)	30.5 ± 46.2	29.2 ± 42.8	29.9 ± 43.4

Continuous variables are presented as means ± standard deviation (SD) unless otherwise indicate; categorical variables are presented as number (percentage)

*Abbreviations*: *BMI*, body mass index; *HDL*, high density lipoprotein; *LDL*, low density lipoprotein; *MET*, metabolic equivalent; *TC*, total cholesterol; *TG*, triglycerides

### Diagnosis of NAFLD

The NAFLD status was measured by abdominal ultrasound in the discovery and prospective internal validation cohorts. Experienced radiologists, blinded to all clinical and laboratory data, qualitatively assessed the liver fat content through a Doppler sonography machine (Sonoscape SSI-5500, Shenzhen, China) with a 3.5-MHz probe and evaluated the degree of steatosis semiquantitatively (0 = absent, ≥ 1 = present) based on hepatorenal echo contrast, deep attenuation, liver brightness, and vascular sharpness according to the guidelines for the diagnosis of non-alcoholic fatty liver diseases in China [23]. We excluded other causes resulting in fatty liver diseases, such as secondary viral hepatitis, drug-induced liver disease, total parenteral nutrition, Wilson’s disease, or excessive alcohol consumption (> 140 g/week for men, > 70 g/week for women). One hundred participants were repeatedly evaluated via ultrasound among the operators and 34 participants were selected for validity evaluation with computed tomography (CT) examinations; both showed good agreement (Spearman’s *r* = 0.911 and 0.905, *P* < 0.001) [24].

The quantified liver fat and volume in the internal validation cohort were detected via hepatic MRI screening with magnetic resonance tomography 1.5 T MAGNETOM Aera and 3 T MAGNETOM Skyra (Siemens Healthcare, Erlangen, Germany). This procedure was conducted by the Universal Medical Imaging Diagnostic Center (Guangzhou, China). With participants under fasting conditions and in the supine position, two MRI techniques were used according to the training and instructions provided by the manufacturers. Two trained image analysts evaluated the scanned images and calculated the proton density fat fraction (PDFF), and NAFLD was defined as a PDFF > 5% [25, 26].

### Stool sample collection and DNA extraction

Stool samples were collected in a sterile container from participants in all cohorts at face-to-face follow-up [19]. The stool specimens were then stored at − 80 °C and transported to the laboratory within 4 h until further DNA extraction. Faecal bacterial DNA was extracted with a QIAamp® DNA Stool Mini Kit (Qiagen, Hilden, Germany) following the manufacturer’s instructions and subsequently stored at − 20 °C for later library construction, which was constructed by amplification of the extracted RNA via two polymerase chain reaction (PCR) procedures. The first PCR amplification of the V3-V4 hypervariable region of the 16S rRNA gene was carried out with a mixture of samples, 1 × KAPA HiFi Hotstart ReadyMix, primers 341F (CCTACGGGNGGCWGCAG) and 805R (GACTACHVGGGTATCTAATCC) in a T100 PCR thermocycler (Bio-Rad). The product was then purified by first fixing it with magnetic beads, eluting impurities with alcohol, and resolubilizing it with nuclease-free water. The purified products were added to HiFi Hotstart ReadyMix and a barcode for the second PCR and then purified as the first purification. The purified amplicons were quantified using a Qubit quantification system (Thermo Scientific, Wilmington, DE, US). Samples meeting the post-PCR quantification minimum (1.6 ng/μL) were pooled in equimolar amounts and advanced for sequencing. The library was sequenced on the Illumina MiSeq platform (Illumina Inc, CA, USA) and paired-end reads of 2 × 250 bp were generated. Quality control (QC) and quality assurance (QA) metrics are maintained for all sample handling, processing, and storage procedures.

### 16S ribosomal RNA (16S rRNA) gene sequencing

Based on the Earth Microbiome Project (EMP) 16S rRNA gene Illumina Amplicon library preparation methodology [27], paired-end reads were assigned to a sample based on their unique barcodes after truncating the barcode and primer sequence, after which they were assembled into single contiguous sequences (contigs). Contigs shorter than 60 bp or containing ‘N’ were screened out, and other high-quality contigs with 97% nucleotide sequence identity or more were clustered as operational taxonomic units (OTUs) via the UPARSE pipeline8 (usearch v8.0.1517). OTU annotations were obtained by using the Ribosomal Database Project (RDP) classifier. Since the RDP classifier provides taxonomic classification only at the genus level, the NCBI collection of completed bacterial genomes was also compared to those sequences to infer species-level taxonomy.

### Faecal metabolome profiling

The extraction of faecal metabolites from stool samples was conducted in line with prior research [19]. We performed metabolic profiling of human faecal samples from the discovery cohort. Faecal metabolites were obtained by targeted metabolomics ultra-performance liquid chromatography with tandem mass spectrometry (UPLC–MS/MS). The platform provides measurements of 198 faecal metabolomes, including 15 subclasses. The frozen faecal samples stored at − 80 °C were thawed on ice to minimize degradation of metabolites. Ten milligrams of the sample was mixed thoroughly with 25 μL of nuclease-free water on a vortex mixer, followed by the addition of 185 μL of cold acetonitrile-methanol (8:2, v/v) for mixing and centrifugation to extract the metabolite samples. Thirty microlitres of the supernatant was then derivatized with 20 μL of derivative reagent on a Biomek 4000 workstation (Biomek 4000, Beckman Coulter, Inc., Brea, CA, USA). After derivatization, the sample was diluted with ice-cold 50% methanol and stored at − 20 °C for 20 min and subsequently centrifuged. The supernatant was mixed with internal standard compounds in a 96-well plate. Serial dilutions of the derivatized stock standards were added to the left wells. A UPLC–MS/MS system (ACQUITY UPLC-Xevo TQ-S, Waters Corp., Milford, MA, USA) was used for analysis, and the QuanMET software (v2.0, Metabo-Profile, Shanghai, China) was used to process the raw UPLC–MS/MS data for peak integration, calibration, and quantification of each metabolite.

### Machine learning algorithms

First, we performed the analyses in parallel using all four commonly used ML algorithms (RF, SVM, logistic regression, and lightGBM) to compare the performances of the different algorithms. The best performing model (LightGBM) was subsequently used to predict essential features of NAFLD based on 272 metadata variables from the discovery cohort. Second, participants of the discovery cohort were randomly grouped into training and test sets at a ratio of 8:2. The training set was subsequently used to construct the initial model by iterating 5000 times and employing tenfold cross-validation. Based on the performance during cross-validation, we applied the optimal parameter combination from the validation set to the final model. Receiver operating characteristic (ROC) curves were constructed, and the area under the receiver operating characteristic (AUC) was calculated to evaluate the model performance. The hyperparameters were tuned to optimize the model. The variables in the validation cohort were imported into the optimized model to estimate the extrapolation of the model.

### SHAP value construction and microbiome risk score (MRS) calculation

The Shapley additive explanation (SHAP) was applied to reveal the LightGBM algorithm results by quantifying the impact of each feature on the model prediction. SHAP values ranged from − 1 to 1, with values greater than 0 indicating a promoting effect on NAFLD and vice versa [28]. A SHAP summary plot was first drawn to visualize the top 20 features contributing to NAFLD in patients with lightGBM, and to reveal the contributions of these features, a SHAP importance matrix plot was generated to depict the importance of each identified feature contributing to NAFLD. The SHAP dependence plots of selected features were created to investigate how each feature influences the presence of NAFLD, presented as the SHAP value > 0 [29].

The microbiome risk score (MRS) was calculated according to the SHAP values of the gut microbiota selected by the LightGBM. The formula is as follows:

$${MRS}_{i}=\sum_{j=1}^{n}{s}_{ij}{s}_{ij}=\left\{\begin{array}{c}0, if shap ij \le 0\\ 1, if shap ij >0\end{array}\right.$$

in which *i* represents individual *i*, *MRS*_*i*_ refers to the MRS of the *i*th individual, *j* represents the *j*th gut microbiota, and ${s}_{ij}$ refers to the MRS for the *j*th gut microbiota in the *i*th individual. The feature set contribution was computed by summing the MRS values per category. The higher the MRS is, the greater the risk of NAFLD. The median of MRS serves as a threshold for measuring the contribution of the microbiota to NAFLD incidence, in which values above the median indicate an elevated contribution to NAFLD and vice versa.

### Gut microbiota transplantation

Distinct and representative stool samples were randomly collected from donors with low MRS, with high MRS but without NAFLD, or with high MRS accompanied with NAFLD in the discovery cohort. Detailed descriptions of faecal sample preparation and faecal microbiota transplantation (FMT) are provided in Additional file 1.

The experimental protocol was approved by the Animal Use and Care Committee at the Medical College, Jinan University.

### Statistical analysis

Both ML and feature selection were conducted using Python 3 and Scikit-Learn (version 0.21.2). Statistical analyses were performed using SPSS version 25.0 (IBM Corp., Armonk, NY, USA), and visualization of the graphs was performed with R version 4.1.3 (www.r-project.org). A *P*-value < 0.05 was considered to indicate statistical significance.

Continuous variables are expressed as the mean ± SD, while categorical variables are shown as numbers (proportions). Multivariate logistic regression was used to test the associations between the presence of NAFLD and the MRS and selected gut microbiota features selected by the LightGBM in the discovery, internal, and prospective cohorts. Model 1 was a crude model, and model 2 was first adjusted for sex and age. Model 3 was further adjusted for cofounders in model 2 plus marital status, education, income, smoking status, alcohol status, tea drinking status, and total energy intake; model 4 was additionally adjusted for body mass index (BMI). Odds ratios (ORs) and corresponding 95% confidence intervals (95% CIs) are expressed per 1 unit of MRS or per-1 SD change in the gut microbiota. The subjects were also divided into four quartiles (Q1–Q4) according to the MRS and multivariable logistic regression was performed across MRS quartiles, with the lowest quartile (Q1) serving as the reference group. Spearman correlation analysis was used to examine the relationship between host faecal metabolites and MRS or selected gut microbiota constituents in the discovery cohort.

The screened differentially abundant metabolites were analysed via MetaboAnalyst 4.0 (https://www.metaboanalyst.ca/) [30]. Prior to analysis, relative peak intensity data were log transformed and normalized to reduce variance between the samples. Principal component analysis (PCA) based on the Bray–Curtis distance was first applied to reduce the unsupervised dimensions of metabolites to profile the global metabolites of the samples. Student’s *t*-test (*P* < 0.05) and Benjamini–Hochberg adjusted false discovery rate (FDR < 0.1) were used to initially identify significant metabolite changes among groups (higher MRS vs. lower MRS; NAFLD vs. controls) [31]. To determine the significant difference in metabolites (SDMs), fold change (the ratio of expression in metabolites between NAFLD patients/higher MRS and control group/lower MRS) > 2 or < 0.5 was used as a screening criterion for SMDs.

## Results

### Study design and characteristics

The details about the selection and number of participants and analyses for the discovery cohort and three validation cohorts are presented in the flow chart in Fig. 1B, with demographic and clinical characteristics shown in Table 1. The discovery cohort included 1546 people, with a mean age of 64.9 ± 5.9 years and 68.4% females. NAFLD was detected in 56.1% of these participants. Compared to those in the discovery cohort, 377 and 747 people in the internal and prospective validation cohorts tended to be younger (65.0 and 64.6 years) and less likely to have NAFLD (33.4% and 35.5%), respectively; moreover, the distributions of sex, marital status, and education were similar among these cohorts.

### Model performance and identification of important features

**Fig. 2:**
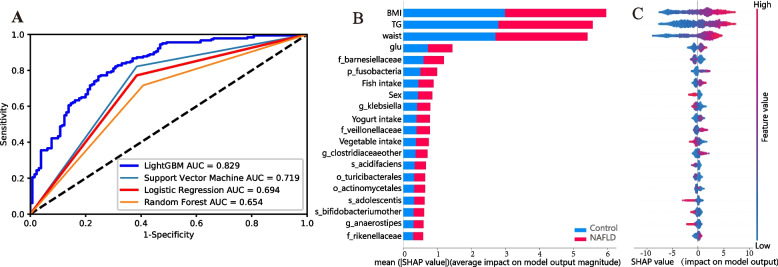
Results based on machine learning model output. **A** Evaluation of the four machine learning algorithms based on the area under the curve AUC of the ROC curve. **B** Importance matrix plot of the top 20 features selected based on LightGBM machine learning algorithms and SHAP value, showing the relative contribution of each variable to NAFLD. **C** SHAP summary plot of the top 20 features selected based on LightGBM machine learning algorithms and SHAP values, in which one dot per individual per feature is coloured in accordance with an attribution value, with red denoting a greater value and blue denoting a lower value. A higher SHAP value indicates a greater risk of NAFLD

Figure 2A shows that the ROC curve showed that the lightGBM model had the best performance and achieved the highest AUC 0.829 in the discovery cohort, followed by the support vector machine (AUC = 0.719), logistic regression (AUC = 0.694), and random forest (AUC = 0.654) models. With respect to the adjusted discovery cohort and the other three validation cohorts, the LightGBM model had the highest predictability of all the trained deep-learning-based segmentation models, with AUCs between 0.762 and 0.984. Metrics for all four models in the four cohorts are provided in Additional file 2: Table S1. A total of 272 features were gathered, after which the top 20 predictive variables were selected by the LightGBM machine learning algorithm, and these selected features reached an AUC of 0.815, indicating that the predictive capacity of these features was similar to that of the overall 272 inputted features (AUC = 0.829) (Additional file 2: Table S2).

Using the importance matrix plot, we identified the top 20 features that had the greatest impact on the prediction power of the model. As shown in Fig. 2B, the three features associated with the highest SHAP values for NAFLD risk were body mass index (BMI), total triglyceride (TG) level, waist, fasting glucose (Glu) level, sex, 12 taxa-related features of the microbiota, and three food intake parameters (fish, yogurt, and vegetable intake). Figure 2C shows that the greater the SHAP values for BMI, TG concentration, waist circumference, glutamate intake, fish intake, and two microbial features (*f_veillonellaceae* and *g_clostridiaceaeother*) were, the greater the likelihood of NAFLD. Conversely, NAFLD was less likely to be detected when SHAP values for male sex, vegetable intake, and five microbial features (*g__klebsiella*,* s__acidifaciens*,* s__adolescentis*,* s__bifidobacteriumother*, and *g__anaerostipes*) increased. Additionally, there were low-to-moderate intercorrelations among a majority of the 12 selected taxa-related characteristics (Additional file 2: Fig. S1).

Several optimal thresholds of these 20 selected features are shown in the SHAP dependence plot, which was used to evaluate the marginal effect of the identified features on the prediction power of the LightGBM (Additional file 2: Fig. S2). We noticed that higher values for BMI, TG level, waist circumference, glucose, fish intake, and higher abundances of four microbiota (*p__fusobacteria*, *f__veillonellaceae*,* g__clostridiaceaeother*, and *f__rikenellaceae*) might contribute to a greater NAFLD risk, whereas female, elevated vegetable intake, and higher abundances of *f__barnesiellaceae*,* g__klebsiella*,* s__acidifaciens*,* o__turicibacterales*,* s__adolescentis*,* s__bifidobacteriumother*, and *g__anaerostipes*, could reduce NAFLD risk.

### Calculation of the MRS and its association with NAFLD

Based on 12 selected microbial features, we calculated the MRS (range 0–12) to evaluate the individual microbiome risk for NAFLD development (Table 2). According to our logistic regression analysis, there was a significant negative association between male sex and the MRS in the discovery cohort (coefficient *r* =  − 0.649; OR = 0.52 [95% CI 0.38, 0.71]; *P* < 0.001). In contrast, BMI and waist circumference were both positively associated with MRS (Additional file 3: Table S1), and the coefficient and odds ratio (OR) (95% CI) were 0.309 and 1.36 (1.25, 1.48; *P* < 0.001) for BMI and 0.082 and 1.09 (1.06, 1.11; *P* < 0.001) for waist circumference, respectively (Additional file 3: Table S1).

**Table 2:** List of components included in the microbiomes risk score (MRS) construction

Microbiome	Taxa annotation
f__barnesiellaceae	k__Bacteria; p__Bacteroidota; c__Bacteroidia; o__Bacteroidales; f__Barnesiellaceae
p__fusobacteria	k__Bacteria; p__Fusobacteria
g__klebsiella	k__Bacteria; p__Proteobacteria; c__Gammaproteobacteria; o__Enterobacteriales; f__Enterobacteriaceae; g__Klebsiella
f__veillonellaceae	k__Bacteria; p__Firmicutes; c__Clostridia; o__Clostridiales; f__Veillonellaceae
g__clostridiaceaeother	k__Bacteria; p__Terrabacteria group; c__Firmicutes; o__Clostridia; f__Eubacteriales; g__Clostridiaceaeother
s__acidifaciens	k__Bacteria; p__Bacteroidetes; c__Bacteroidia; o__Bacteroidales; f__Bacteroidaceae; g__Bacteroides; s__acidifaciens
o__turicibacterales	k__Bacteria; p__Firmicutes; c__Bacilli; o__Turicibacterales
o__actinomycetales	k__Bacteria; p__Actinobacteria; c__Actinobacteria; o__Actinomycetales
s__adolescentis	k__Bacteria; p__Actinobacteria; c__Actinobacteria; o__Bifidobacteriales; f__Bifidobacteriaceae; g__Bifidobacterium; s__adolescentis
s__bifidobacteriumother	k__Bacteria; p__Terrabacteria group; c__Actinobacteria; o__Actinomycetia; f__Bifidobacteriales; g__Bifidobacteriaceae; s__Bifidobacteriumother
g__anaerostipes	k__Bacteria; p__Firmicutes; c__Clostridia; o__Clostridiales; f__Lachnospiraceae; g__Anaerostipes
f__rikenellaceae	k__Bacteria; p__Bacteroidetes; c__Bacteroidia; o__Bacteroidales; f__Rikenellaceae

The MRS is generated based on these 12 microbiome features

*Abbreviation*: *MRS*, microbiomes risk score

**Fig. 3:**
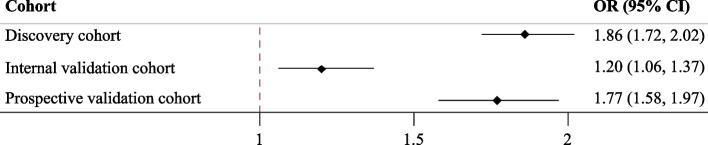
Association of the microbiome risk score (MRS) with NAFLD risk in different cohorts Note: Logistic regression was used to estimate the odds ratio (OR) and 95% confidence interval (CI) of NAFLD per one unit change in the MRS, adjusting for age, sex, marital status, education, income, smoking status, drinking status, tea status, and total energy intake

According to our logistic regression analysis, a 1-unit change in the MRS was positively associated with an increase in odds of NAFLD in the discovery cohort (crude OR = 1.85 [95% CI 1.71, 1.99]; *P* < 0.001). This association persisted in model 3 which was adjusted for most potential predictors, including age, sex, marital status, education, income, smoking status, drinking status, tea status, and total energy intake (OR = 1.86 [95% CI 1.72, 2.02]; *P* < 0.001). Similar results were also then observed in the internal validation cohort, in which the crude and multivariate ORs and their corresponding 95% CIs were 1.21 (1.07, 1.36; *P* = 0.003) and 1.20 (1.06, 1.37; *P* = 0.004), respectively; in the prospective validation cohort, the crude and multivariate ORs and their corresponding 95% CIs were 1.73 (1.56, 1.92; *P* < 0.001) and 1.77 (1.58, 1.97; *P* < 0.001), respectively (Fig. 3; Additional file 3: Table S2). According to the sensitivity analysis, the positive association between the MRS and the incidence of NAFLD remained significant in the discovery and internal validation cohorts in Model 4, which was further adjusted for BMI, suggesting the robustness of the results.

According to multivariate logistic regression using MRS quartiles, when setting Q1 of the MRS as a reference, higher MRS quartiles were positively associated with an elevated incidence of NAFLD in the discovery cohort across the four models, and the ORs and 95% CIs of Q4 in four models were 11.98 (8.60, 16.70), 18.86 (12.53, 28.40), 11.93 (8.56, 16.63), and 16.92 (11.37, 25.17) (all *P* values < 0.001). In agreement with the results of the discovery cohort, individuals in the highest quartile of the MRS in the internal validation cohort had significantly elevated likelihood of NAFLD (model 1: OR = 3.50 [95% CI = 1.83, 6.71]; model 2: OR = 3.44 [95% CI = 1.72, 6.86]; model 3: OR = 3.52 [95% CI = 1.83, 6.75]; model 4: OR = 3.47 [95% CI = 1.75, 6.90]; all *P* values < 0.001). These positive associations between the MRS and NAFLD incidence were further validated in the prospective validation cohort (model 1: OR = 35.97 [95% CI = 16.00, 80.95]; model 2: OR = 42.64 [95% CI = 18.28, 99.46]; model 3: OR = 11.93 [95% CI = 8.56, 16.63]; model 4: OR = 16.92 [95% CI = 11.37, 25.17]; all *P* values < 0.001) (Additional file 3: Table S3).

### The relationship between selected gut microbiota features and NAFLD

**Fig. 4:**
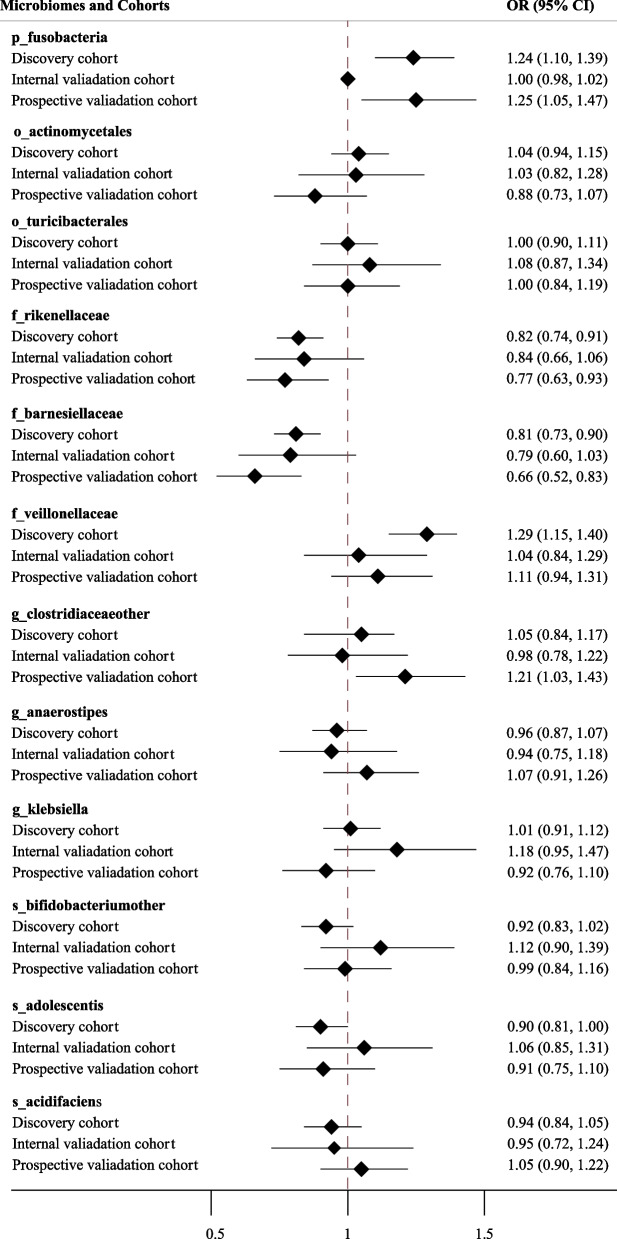
Association of the microbiomes selected through machine learning with NAFLD risk in different cohorts. Note: Logistic regression was used to estimate the odds ratio (OR) and 95% confidence interval (CI) of NAFLD incidence per SD change in the microbiota, adjusting for age, sex, marital status, education, income, smoking status, drinking status, tea status, and total energy intake

In terms of the relationships between the 12 identified microbiotal features and NAFLD, we found that a per SD change in the abundance of *f__veillonellaceae* was positively associated with a greater likelihood of NAFLD, and OR and 95% CIs were 1.29 (1.15, 1.44). Conversely, lower odds of NAFLD were detected in individuals with a greater abundance of *f__rikenellaceae* (OR = 0.81 [95% CI 0.74, 0.88]), *f__barnesiellaceae* (OR = 0.78 [95% CI 0.71, 0.85]), *s__adolescentis* (OR = 0.92 [95% CI 0.84, 1.00]) (Fig. 4; Additional file 4: Table S1); however, when the microbiota was treated as a binary variable according to the SHAP value, 12 microbiota were significantly associated with NAFLD (Additional file 4: Table S2 and Fig. S1)*.*

### Identification of significantly different metabolites (SDMs)

The PCA score plot of the faecal metabolites of the two groups (MRS > 5 vs. MRS ≤ 5) in the discovery cohort is shown in Additional file 5: Fig. S1. PC1 and PC2 explained 26.1% and 6.4%, respectively, of the total variance. We further visualized the distribution of 101 significant metabolites (Student’s *t*-test *P* < 0.05, FDR < 0.1) in Additional file 5: Fig. S2. Moreover, volcano plot analysis revealed that one downregulated (*picolinic acid*) and three upregulated metabolites (*glycocholic*,* D_Maltose and alpha_lactose*, and *taurocholic acid*) were significantly differentially expressed (FDR < 0.10; Additional file 5: Fig. S3, Table S1). Among these genes, the *picolinic acid* had a negative log_2_ FC (− 1.625 with FDR = 0.056). Among the three upregulated candidate metabolites, *D_Maltose and alpha_Lactose* had the highest log_2_ FC (1.197 with FDR = 0.004), followed by *glycocholic acid* (log_2_ FC = 1.160 with FDR = 0.003) and *taurocholic acid* (log_2_ FC = 1.104 with FDR = 0.028) (Additional file 5: Table S1).

Subsequently, we used the same method as above to identify SDMs between patients with and without NAFLD; one differentially abundant metabolite was found to be *taurocholic acid* (log_2_ FC = 1.278 with FDR = 0.066; Student’s *t*-test: *P* = 0.017, FDR = 0.066; Additional file 5: Table S2 and Fig. S4-6). Combining the above significant differentially abundant metabolites identified among MRS and NAFLD status groups, *taurocholic acid* was ultimately recognized as a common differentially abundant metabolite in our study.

### Taurocholic acid was significantly associated with selected gut microbiota features

We further analysed the association of *taurocholic acid* with 12 selected gut microbiota (Additional file 5: Table S3). We noticed that 7 out of the 12 gut microbiota constituents were significantly correlated with *taurocholic acid*, among which there were significant positive correlations with *p__fusobacteria* (coefficient *r* = 0.198) and *g__clostridiaceaeother* (coefficient *r* = 0.099), while they were inversely correlated with *o__actinomycetales* (coefficient *r* =  − 0.142), *o__turicibacterales* (coefficient *r* =  − 0.070), *f__barnesiellaceae* (coefficient *r* =  − 0.357), *f__rikenellaceae* (coefficient *r* =  − 0.345), and *s__adolescentis* (coefficient *r* =  − 0.085).

### Mouse experiment validation

To evaluate the role of gut microbiota features in NAFLD pathogenesis, we applied an FMT mouse model of NAFLD. As shown in Additional file 1: Fig. S1 (A), the trend towards body weight gain was more evident in the high MRS + NAFLD group and high MRS + non-NAFLD group than in the low MRS and control (only HFD) groups. Compared with those in the low MRS group, the body weight, liver weight, Lee’s obesity index, NAS score, and hepatic TG content increased in the high MRS + NAFLD and high MRS + non-NAFLD groups (Additional file 1: Fig. S1 B-F). According to the HE staining results, compared with those in the control group, the stool samples from both the high MRS + NAFLD and high MRS + non-NAFLD groups had more lipid droplets in the cells and exhibited a greater extent of liver steatosis. In contrast, lipid accumulation within liver tissues was alleviated in the low MRS group (Additional file 1: Fig. S1 G).

## Discussion

In this study, we identified 12 gut microbial taxa associated with NAFLD by using an interpretable ML algorithm and observed significant associations between NAFLD and gut microbiota signatures. Overall, we found that a greater abundance of *p__fusobacteria* and *f__veillonellaceae* might contribute to the development of NAFLD, while *f__rikenellaceae*, *f__barnesiellaceae*, and *s__adolescentis* might reduce the occurrence of NAFLD. We further calculated the MRS according to our prior study [19] and found that a higher MRS was positively associated with a greater incidence of NAFLD; this MRS-NAFLD association was successfully replicated in the internal and prospective validation cohorts. We identified a faecal metabolite of bile acids (*taurocholic acid*), which was positively correlated with a higher MRS and a greater risk of NAFLD. Moreover, the FMT experiment further demonstrated that stool samples from participants with a higher MRS could significantly exaggerate the increase in body weight, liver weight, hepatic TG content, Lee’s index, NAS, and the extent of liver steatosis.

Many epidemiological studies have reported alterations in the gut microbiome composition among individuals with and without NAFLD, but the evidence has remained inconclusive. For the relative abundance of pathogenic bacteria, a case–control study involving 25 NAFLD patients and 22 healthy subjects in Shanghai demonstrated that the abundance of *fusobacteria* was significantly greater in NAFLD patients than in the control group (*P* < 0.01) [32]. Animal models have consistently shown an elevated abundance of *fusobacteria* in rats with HFD-induced NAFLD [33], or in mice fed a high-fat choline-deficient diet for 18 weeks [34]. This positive association might be partly explained by an increase in microbial gut toxins due to pathogens derived mainly from the *Fusobacteria phylum*, thus disrupting the balance of energy metabolism [32]. However, the decreased bacterial diversity and relative abundance of *fusobacteria* were observed in mice with NAFLD induced by a 6-month western diet (enriched with fat, sugar, and sucrose chow) [35]. Thus, further large-scale studies with homogenous patient cohorts and standardized methods are warranted to explore the effect of *fusobacteria* on the development of NAFLD.

In line with our findings, a previous case–control study showed that an increased abundance of *Veillonellaceae* was one of the most common changes reported in NAFLD patients [36]. An animal study further demonstrated an elevated abundance of *Veillonellaceae* in rats with HFD- induced NAFLD [37]. In addition, after taking stool samples from 129 NAFLD and 75 non-NAFLD individuals fed a high- carbohydrate diet, a Korean study revealed that the *Veillonellaceae* could be regarded as one of the most vital microbial taxa and could significantly improve the predictive value of NAFLD in the total population using a random forest model [38]. The *Veillonellaceae* is a lactate-degrading microbe that obtains its carbon primarily from lactate [39]; it can produce endotoxin and cause immunoreaction [40], and the enrichment of *Veillonellaceae* is also associated with metabolic syndrome [41]. Moreover, *Veillonellaceae* might be one of the contributing factors to fibrosis severity because it was found to be positively associated with serum-free fatty acids and significantly correlated with adipose tissue insulin resistance and glycosylated haemoglobin in nonobese NAFLD patients [15]. Hence, *Veillonellaceae* might be used as a diagnostic marker in the NAFLD population.

In contrast, we noticed inverse associations between NAFLD and three gut microbiota, namely, *f__rikenellaceae*, *f__barnesiellaceae*, and *s__adolescentis.* Consistent with our findings, a matched case–control study reported a reduced proportion of *Rikenellaceae* in NAFLD patients, compared to healthy controls [42]. An animal study by Chen H et al. [43] showed that *Rikenellaceae* was negatively associated with the serum parameters glucose, insulin, and lipid metabolism (triglycerides, TG; total cholesterol, TC) and liver function parameters (alanine aminotransferase, ALT; aspartate transaminase, AST) but was positively associated with improved glucose tolerance and insulin sensitivity in HFD-induced NAFLD mice. The above evidence indicated that *Rikenellaceae* might effectively contribute to the balance between energy harvesting and hepatic metabolism, thereby inhibiting the development of NAFLD.

The abundance of the family *barnesiellaceae* was lower in NAFLD patients than in non-NAFLD patients in the present study. A recent study consistently revealed a decreased abundance of *Barnesiella* among NAFLD patients, compared to that in control and drug-induced liver injury groups [44]. This might be partly ascribed to its association with the production of short-chain fatty acids (SCFAs) [45], which might exert a beneficial effect against NAFLD progression [46]. However, notably few studies on the association between the abundance of *Barnesiellaceae* and NAFLD exist. We hypothesize that this microbiota signature could be negatively associated with NAFLD and could be regarded as s microbiota-derived signature for reducing the possible development of NAFLD. With respect to *s__adolescentis*, we noticed that the gut microbiota was enriched in the non-NAFLD. A case–control study involving 75 children also indicated that the abundance of *adolescentis* was significantly decreased in NAFLD patients [47]. This microbial signature was also found to relieve NAFLD by increasing the concentration of SCFAs in the intestines of mice [48]. Furthermore, *adolescentis* is an anti-inflammatory agent, and probiotics inhibit inflammation, regulate lipid metabolism, and alleviate NAFLD by increasing fibroblast growth factor 21 (FGF21) sensitivity [49, 50]. A*dolescentis* can reduce nuclear factor-kappa B (NF-κB) activation in an intestinal cell line (Caco2), limit the production of lipopolysaccharide (LPS), contribute to the release of SCFAs in the gut, and subsequently alleviate the progression of NAFLD [51, 52].

Our results indicated that a combination of specific gut microbiota (presented as a higher MRS) may be a risk factor for NAFLD, and these findings were further replicated in two validation cohorts, indicating the good predictive performance of our ML model. Although many ML methods are still opaque, the LightGBM model is characterized by its relatively fast speed, high efficiency, and excellent performance [53] because it can reduce the calculation cost of the gain for each split, grow trees leafwise and vertically, and accelerate the training process compared to other decision tree algorithms [54]. A better predictive value of lightGBM after the comparison of the performance across different ML algorithms was reported by prior studies [54, 55] Similarly, our results also demonstrated the excellent performance of LightGBM, which could be superior to the other three algorithms evaluated by us. Additionally, the SHAP value, which represents a predictor’s marginal contribution to the risk of a complication or outcome [56], was widely utilized for interpreting the interpretability of the ML. Microbial signatures with higher SHAP values were more relevant to the prediction of NAFLD. In addition to the specific gut microbial signatures mentioned above, the other seven identified gut microbiota (*g__klebsiella*,* g__clostridiaceaeother*,* s__acidifaciens*,* o__turicibacterales*,* o__actinomycetales*,* s__bifidobacteriumother*, and *g__anaerostipes*) were further taken into consideration to calculate the MRS. A risk score was estimated based on the SHAP value for all 12 identified gut microbes and showed a superior prediction accuracy for T2DM compared with traditional methods [19]. Based on the advanced interpretable machine learning lightGBM and SHAP algorithms, the FMT was conducted, and it further revealed that FMT of stool samples from humans with high MRS with or without NAFLD to germ-free mice could result in elevated levels of body weight, liver weight, Lee’s obesity index, NAS, and hepatic TG content, exacerbating NAFLD, whereas faecal transfer from those with low MRS may inversely reduce the levels of these parameters and alleviate NAFLD symptoms. Collectively, our LightGBM algorithm and animal experiments provided supporting evidence of a causal role for the alteration of the gut microbiota composition in the progression of NAFLD.

Accumulating evidence has shown that the gut microbiota might be involved in the aetiology of NAFLD. Specific faecal microbiota might increase intestinal permeability, which releases lipopolysaccharide (LPS) into the host and subsequently triggers systemic and tissue inflammation, and immunity might be affected by microbial metabolites, such as trimethylamine N-oxide (TMAO), choline, ethanol, and bile acid signalling [2]. To further explore the potential mechanisms of action of faecal metabolites and pathways, we also investigated the metabolomic signatures associated with MRS or gut microbiota features. When combining the associations of MRS and NAFLD with different faecal metabolites, we found that the concentration of the faecal metabolite *taurocholic acid* was positively correlated with a higher MRS and several NAFLD-promoted gut microbiota (*p__fusobacteria* and *g__clostridiaceaeother*). Faecal microbiota dysbiosis is associated with altered bile acid homeostasis [57]. As bile acids are secreted into bile, they can further influence the development and progression of gastrointestinal and liver health by modifying the microbiota, altering the intestinal barrier function and modulating the innate immune system [58]. The bile acid profile of germ-free animals was dominated by taurine-conjugated bile acids (especially *taurocholic* *acid* and *taurolycholic* *acid*) [59]. However, the mechanism by which *taurocholic acid* increases the incidence of NAFLD remains to be elucidated. A prior animal model suggested that high plasma *taurocholic acid* levels may aggravate cholesterol-induced triglyceride accumulation in the human normal immortalized hepatocyte cell line LO2, thus promoting the progression of non-alcoholic steatohepatitis—hepatocellular carcinoma [60]. However, a limited number of studies have investigated the potential role of the gut metabolite of *taurocholic acid* in the development of NAFLD; thus, further investigations are warranted to unravel the mysteries of the associations between these associations.

This study has the following limitations. First, the main results were obtained from several middle-aged and elderly Chinese cohorts; thus, our results might not be generalizable to other ethnic and age groups. Second, the current study did not focus on coexisting fungal or viral communities other than gut bacterial communities. Third, as a result of this limited sample collection technique, several stool samples were not collected at either time point, leading to fewer patients having paired microbiota data. Finally, an analysis of the gut microbiota generally cannot distinguish the biogeography and dynamics of microbial populations in the gastrointestinal tract.

## Conclusions

In summary, this study used the lightGBM-SHAP algorithm to identify gut microbiota features and revealed that the presence of specific gut microbiota (*p__fusobacteria* and *f__veillonellaceae*) was positively associated with NAFLD, but *f__rikenellaceae*, *f__barnesiellaceae*, and *s__adolescentis* were negatively associated with NAFLD. Both observational and experimental data illuminate the contribution of the MRS to the progression of NAFLD. Further large-scale studies with homogenous patient cohorts and standardized methods are necessary to examine the biological relevance and mechanistic insights of gut metabolites (*taurocholic acid*) in NAFLD. Awareness of these associations is important for predicting NAFLD through the gut microbiota.

### Supplementary Information

**Additional file 1. **Supplementary methods for animal experiment and results in Fig. S1.

**Additional file 2. **Details of model performance and identification of important features. Table S1. Metrics for all four models in the three cohorts. Table S2. Comparison of the prediction performance of all inputted and selected features in different cohorts. Fig. S1. The inter-correlation of selected taxa-related features in the discovery cohort. Fig. S2. The marginal effect of individual selected features on non-alcoholic fatty liver disease.

**Additional file 3. **Details of the association between the MRS and NAFLD. Table S1. Associations of baseline adiposity and dietary factors with microbiome risk score (MRS) in discovery cohort. Table S2. Association of the gut microbiome risk score (MRS) with NAFLD. Table S3. Association of the quartiles of gut MRS with NAFLD in the 3 cohorts.

**Additional file 4. **Details of the relationship between selected gut microbiota features and NAFLD. Table S1. Association between selected microbiome features and NAFLD in the 3 cohorts. Table S2. Logistics regression was used to estimate association between NAFLD and selected microbiome features at higher abundance (SHAP value higher than 0) and lower abundance (SHAP value lower than 0). Fig. S1. Logistics regression was used to estimate association between NAFLD and selected microbiome features at higher abundance (SHAP value higher than 0) and lower abundance (SHAP value lower than 0).

**Additional file 5. **Details of identification of significantly different metabolites and the association between SDM with gut microbiota features. Fig. S1. The Principal Component Analysis (PCA) score plot of the first two principal components for metabolite levels among groups by MRS in the discovery cohort. Fig. S2. Univariate analysis results of differential metabolites in different groups by MRS in the discovery cohort. Fig. S3. Important metabolites selected by volcano plot in different groups by MRS in the discovery cohort. Fig. S4. The Principal Component Analysis (PCA) score plot of the first two principal components for metabolite levels among groups by whether with NAFLD or not in the discovery cohort. Fig. S5. Univariate analysis results of differential metabolites in different groups by whether with NAFLD or not in the discovery cohort. Fig. S6. Important metabolites selected by volcano plot in different groups by whether with NAFLD or not in the discovery cohort. Table S1. Four significant different metabolites (SDMs) in faecal samples among discovery cohort (higher MRS group vs. lower MRS group). Table S2. One significant different metabolites (SDMs) in faecal samples among discovery cohort (NAFLD group vs. control group). Table S3. Correlations between selected microbiomes and Taurocholic acid.

## Data Availability

16S rRNA gene sequencing data from the Guangzhou Nutrition and Health Study Nutrition and Health Study (GNHS) have been deposited in in the Genome Sequence Archive (GSA) (https://ngdc.cncb.ac.cn/gsa/) at accession number CRA006769. Other data that support the findings of this study are available upon request from the corresponding author.

## Abbreviations

ALT: Alanine aminotransferase
AST: Aspartate transaminase
AUC: Area under the receiver operating characteristic
BMI: Body mass index
CIs: Confidence intervals
CT: Computed tomography
CVDs: Cardiovascular diseases
EMP: Earth Microbiome Project
FDR: False discovery rate
FFQ: Food Frequency Questionnaire
FGF21: Fibroblast growth factor 21
FMT: Faecal microbiota transplantation
Glu: Fasting glucose
GNHS: Guangzhou Nutrition and Health Study
LPS: Lipopolysaccharide
MET: Metabolic equivalent
ML: Machine learning
MRI: Magnetic resonance imaging
MRS: Microbiome risk score
NAFLD: Non-alcoholic fatty liver disease
NASH: Non-alcoholic steatohepatitis
NF-κB: Nuclear factor-kappa B
OR: Odds ratio
OTUs: Operational taxonomic units
PAQs: Physical activity questionnaires
PCA: Principal component analysis
PCR: Polymerase chain reaction
PDFF: Proton density fat fraction
Q: Quartiles
QA: Quality assurance
QC: Quality control
RDP: Ribosomal Database Project
RF: Random forest
ROC: Receiver operating characteristic
SCFAs: Short-chain fatty acids
SDMs: Significant difference in metabolites
SHAP: Shapley additive explanation
SVM: Support vector machine
T2DM: Type 2 diabetes
TC: Total cholesterol
TG: Triglyceride
TMAO: Trimethylamine N-oxide
UPLC–MS/MS: Ultra-performance liquid chromatography with tandem mass spectrometry
US: Abdominal ultrasound
