# Supplementary material for: Gut microbiome features and metabolites in non-alcoholic fatty liver disease among community-dwelling middle-aged and older adults
Source: BMC Med. 2024 Mar 7;22:104. doi: 10.1186/s12916-024-03317-y (PMC10921631; doi:10.1186/s12916-024-03317-y)
Supplement: Supplementary file 1 — Additional file 1. Supplementary methods for animal experiment and results in Fig. S1. [file 12916_2024_3317_MOESM1_ESM.docx]

**Additional file 1.** **Mouse experiment validation.**

**Experimental design**

***Preparation of faecal suspensions***

Participants in the discovery cohort were classified into three subgroups: i) those with low MRS; ii) those with high MRS but without NAFLD; and iii) those with high MRS accompanied by NAFLD. Three distinct and representative stool samples were randomly collected from each group of donors. The faecal samples were subsequently transported on ice to the laboratory and was processed within 2 h after collection. With 2 g of fresh faeces collected from each selected participant, a total of 6 g of faeces was mixed in each subgroup and then diluted at a ratio of 1:10 using a sterile PBS solution. After thorough vortexing (5 minutes), the diluted faeces were centrifuged at 4 °C 100 rpm/min for 5 minutes. To prevent environmental contamination, the clear supernatants of faecal suspensions were subsequently collected in sterile tubes and stored at -80 °C until use.

***Faecal microbiota transplantation (FMT)***

FMT was carried out based on previous methods ^30^. A total of 40 specific pathogen-free (SPF) mice (C57BL/6J, 7 weeks old, male) were obtained from the Guangdong Medical Laboratory Animal Center and were randomly divided into four groups (10 mice per group) after being adaptively raised for one week. To create pseudo-germ-free mouse models, SPF mice were orally administered broad-spectrum antibiotics (vancomycin: 0.5 g/L, neomycin sulfate 1 g/L, metronidazole 1 g/L, and ampicillin 1 g/L; Macklin, Shanghai, China) dissolved in sterile drinking water for 6 consecutive days ^30,31^. After a one-day washout period, FMT was performed in three groups of mice by orally administering 0.2 ml of 10-fold diluted faecal suspension twice a week for 12 weeks, whereas the last group was only given 0.2 ml of PBS solution for gavage feeding as the control group. The experimental mice were housed under SPF conditions and fed a high-fat diet (HFD) during the whole FMT process. At the end of the experiments, all the mice were weighed; Lee’s obesity index was calculated by the formula (weight ^0.33^ /Naso-Anal length) ^32^, and the NAFLD activity score (NAS) was calculated as the grade for steatosis (grade 0–3), lobular inflammation (grade 0–3), and hepatocellular ballooning (grade 0–2) ^33^.

All the mice were sacrificed and the liver biopsy samples were taken from the dissected mice, and then stained with hematoxylin and eosin (H&E). Liver tissue was fixed in 4% paraformaldehyde (Biosharp, China) for 24 hours, paraffin embedded, sectioned to a thickness of 6 μm, and stained with different concentrations of ethanol and xylene, and dehydration followed by staining of the nuclei with 5% haematoxylin (Leagene, China) solution for 10 minutes. After rinsing in distilled water for 5 minutes, the stained samples were incubated in 0.1% HCl-ethanol for 30 seconds. The samples were then re-stained with eosin solution for 2 minutes before washing and dehydration. Fluorescence microscopy was used for imaging ^34^.

The frozen sections were prepared for stained with Oil-Red-O (Leagene, China) to observe the degree of liver steatosis. Frozen tissue was fixed in 4% formaldehyde for 24 hours, and then dehydrated in a 30% sucrose solution. The embedded samples were stained for 8 min using Oil Red O working solution protected from light after rinsing in evaporated water and then rinsing in distilled water. We observed the staining of samples that were soaked in haematoxylin for 2 min 30 s and then washed in 1% acidic ethanol (Leagene, China) fractionation solution for 1 second. Furthermore, the samples were soaked in tap water for 10 min for rebaining, after which the films were sealed using glycerol gelatine (BBcellProbe, China).

## Figure S1. Validation mouse experiment.


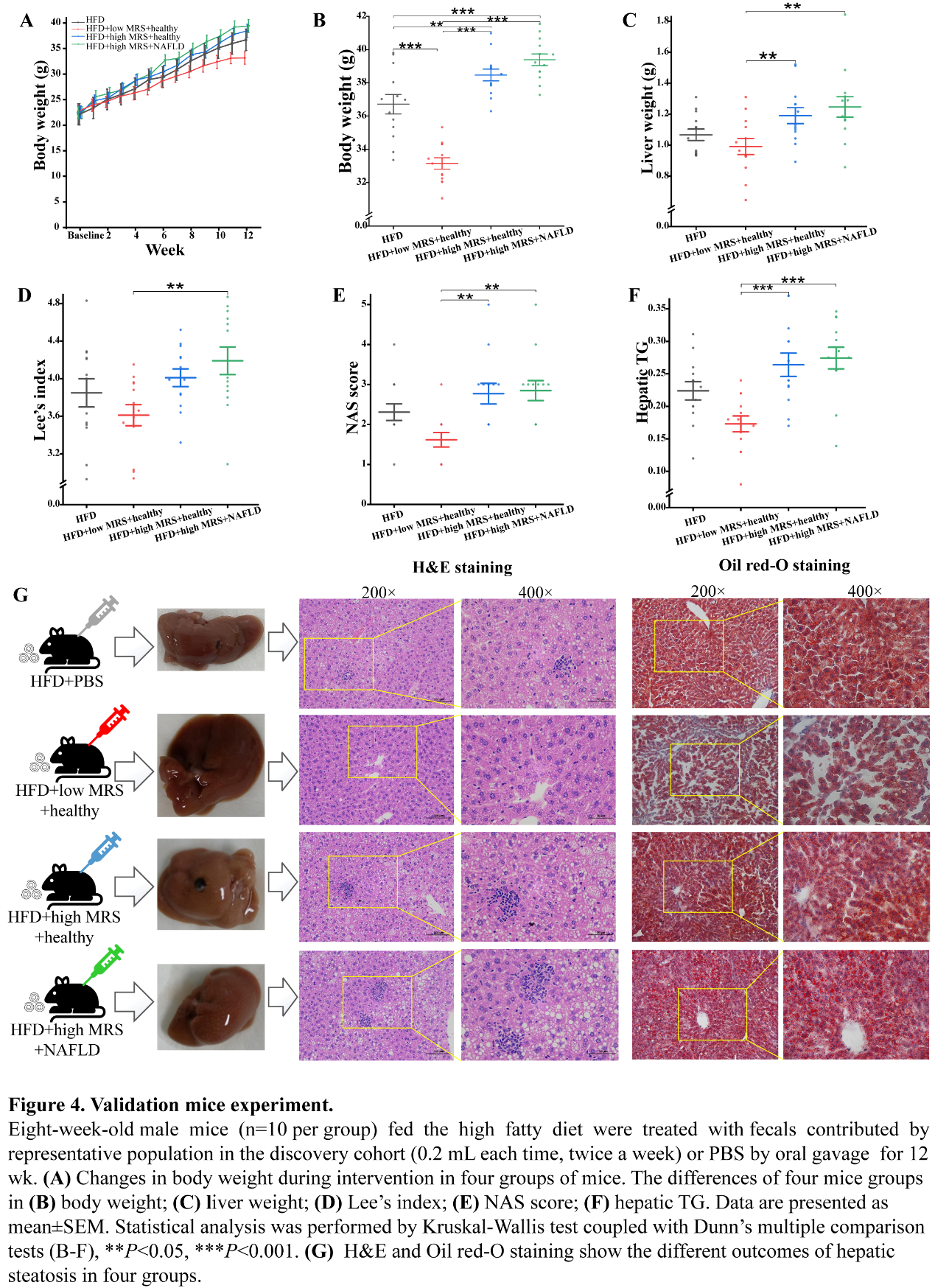


Eight-week-old male mice (n=l0 per group) fed a high fat diet were treated with faeces form a representative population in the discovery cohort (0.2 mL each time, twice a week) or with PBS by oral gavage for 12 weeks. (A) Changes in body weight during the intervention in the four groups of mice. Differences of four mice groups in (B) body weight; (C) liver weight; (D) Lee's index; (E) NAS score; and (F) hepatic TG among the four groups of mice. The data are presented as the mean ±SEMs. Statistical analysis was performed by the Kruskal‒Wallis test coupled with Dunn's multiple comparison test (B-F), **p<0.05, ***p<0.001. (G) H&E and Oil Red O staining show the different outcomes of hepatic steatosis in the four groups.
