# Supplementary material for: Gut microbiome features and metabolites in non-alcoholic fatty liver disease among community-dwelling middle-aged and older adults
Source: BMC Med. 2024 Mar 7;22:104. doi: 10.1186/s12916-024-03317-y (PMC10921631; doi:10.1186/s12916-024-03317-y)
Supplement: Supplementary file 2 — Additional file 2. Details of model performance and identification of important features. Table S1. Metrics for all four models in the three cohorts. Table S2. Comparison of the prediction performance of all inputted and selected features in different cohorts. Fig. S1. The inter-correlation of selected taxa-related features in the discovery cohort. Fig. S2. The marginal effect of individual selected features on non-alcoholic fatty liver disease. [file 12916_2024_3317_MOESM2_ESM.docx]

**Additional file 2.** **Details of model performance and identification of important features**

## Table S1. Metrics for all four models in the three cohorts.

Comparison of the prediction performance of Light GBM, support vector machine, logistic regression and random forest in the discovery cohort, internal validation cohort and prospective validation cohort. Besides, parameters are adjusted in discovery cohort 1.

| **Algorithm** | **Discovery cohort** | **Adjusted in discovery cohort** | **Internal validation cohort** | **Prospective validation cohort** |
| --- | --- | --- | --- | --- |
| Light GBM | 0.829 | 0.839 | 0.791 | 0.987 |
| Support vector machine | 0.735 | 0.767 | 0.656 | 0.699 |
| Logistic regression | 0.694 | 0.771 | 0.688 | 0.697 |
| Random forest | 0.654 | 0.723 | 0.721 | 0.922 |

## Table S2. Comparison of the prediction performance of all inputted and selected features in different cohorts.

| **Features** | **AUC** |
| --- | --- |
| 272 features | 0.829 |
| Identified 20 features | 0.815 |

**Abbreviations**: AUC, area under the curve.

## Fig. S1. The inter-correlation of selected taxa-related features in the discovery cohort.


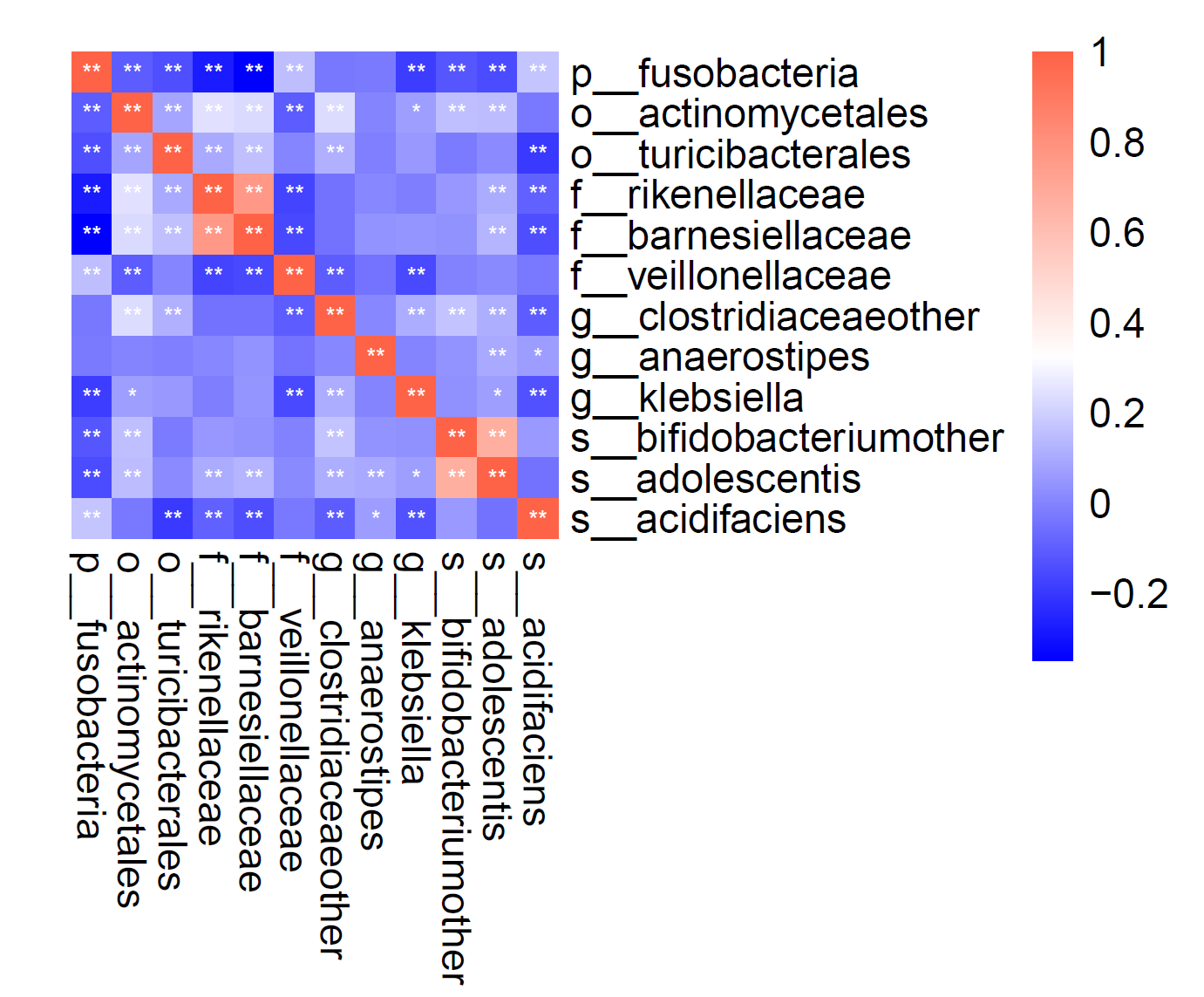


## Fig. S2. The marginal effect of individual selected features on non-alcoholic fatty liver disease.


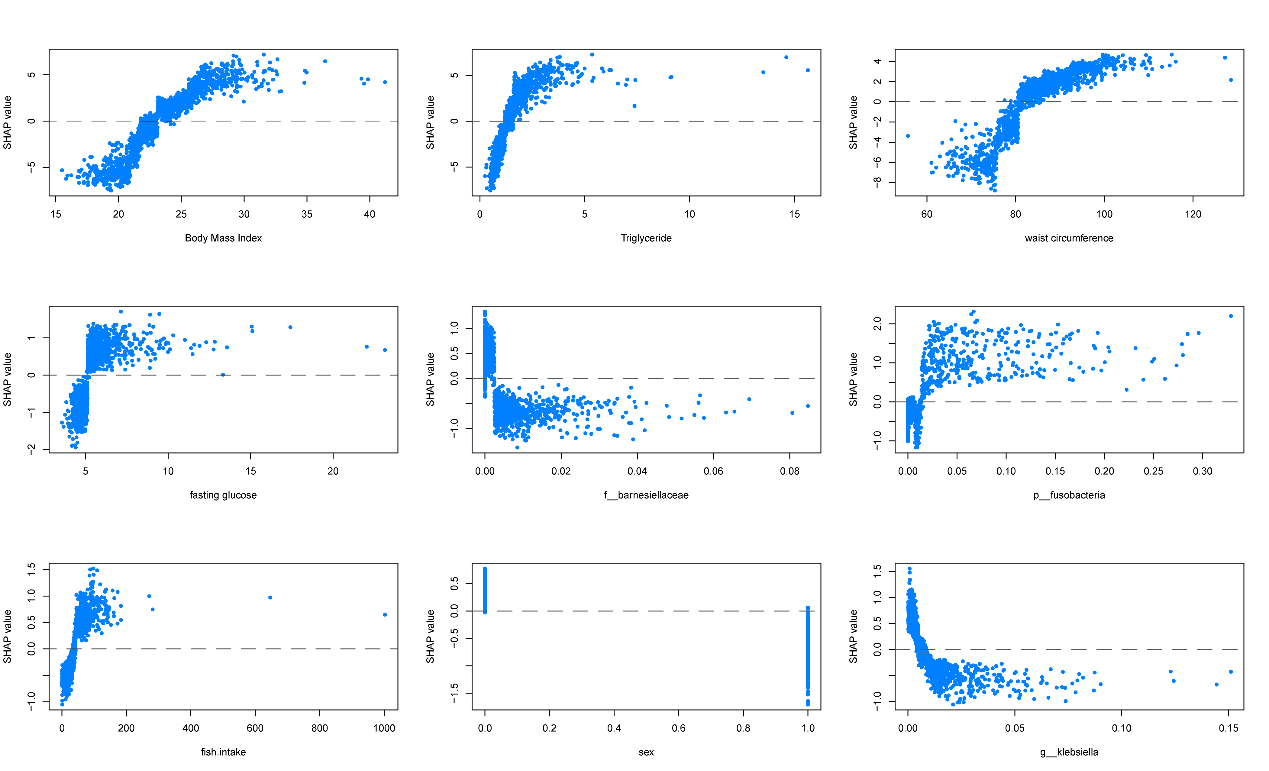

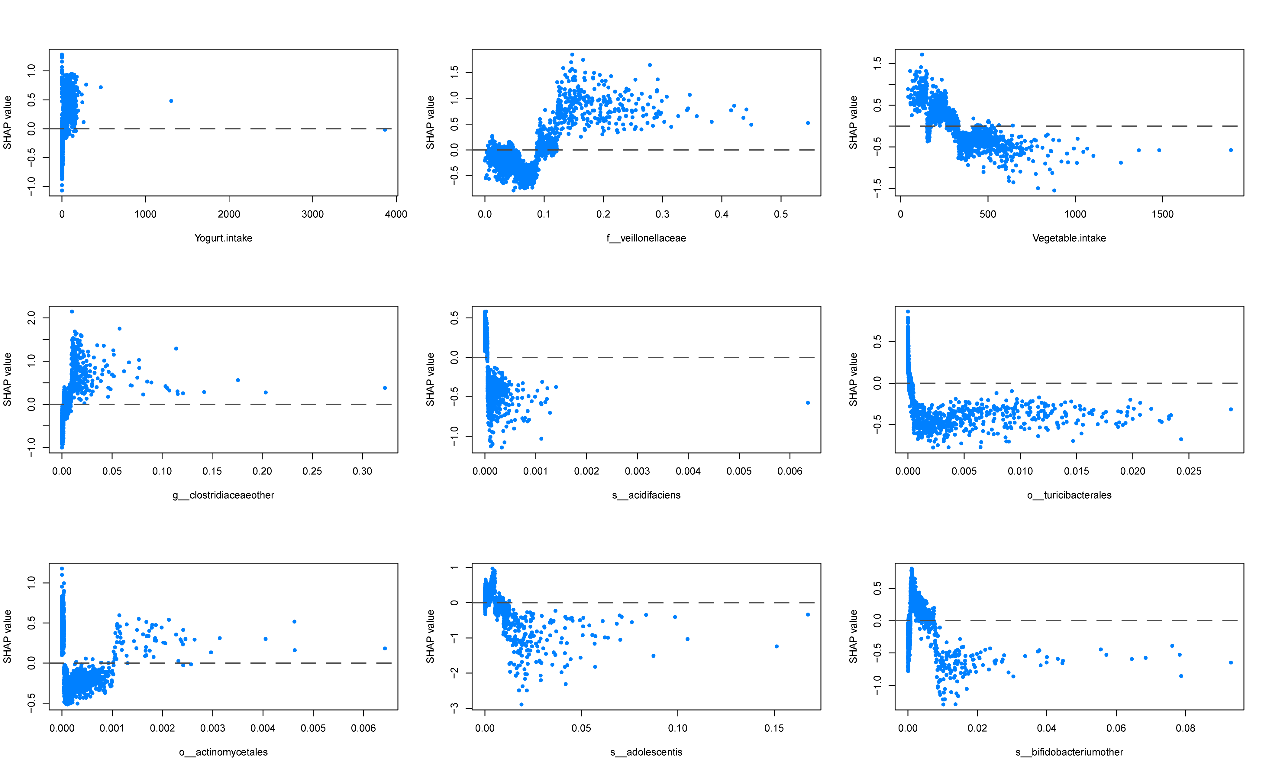

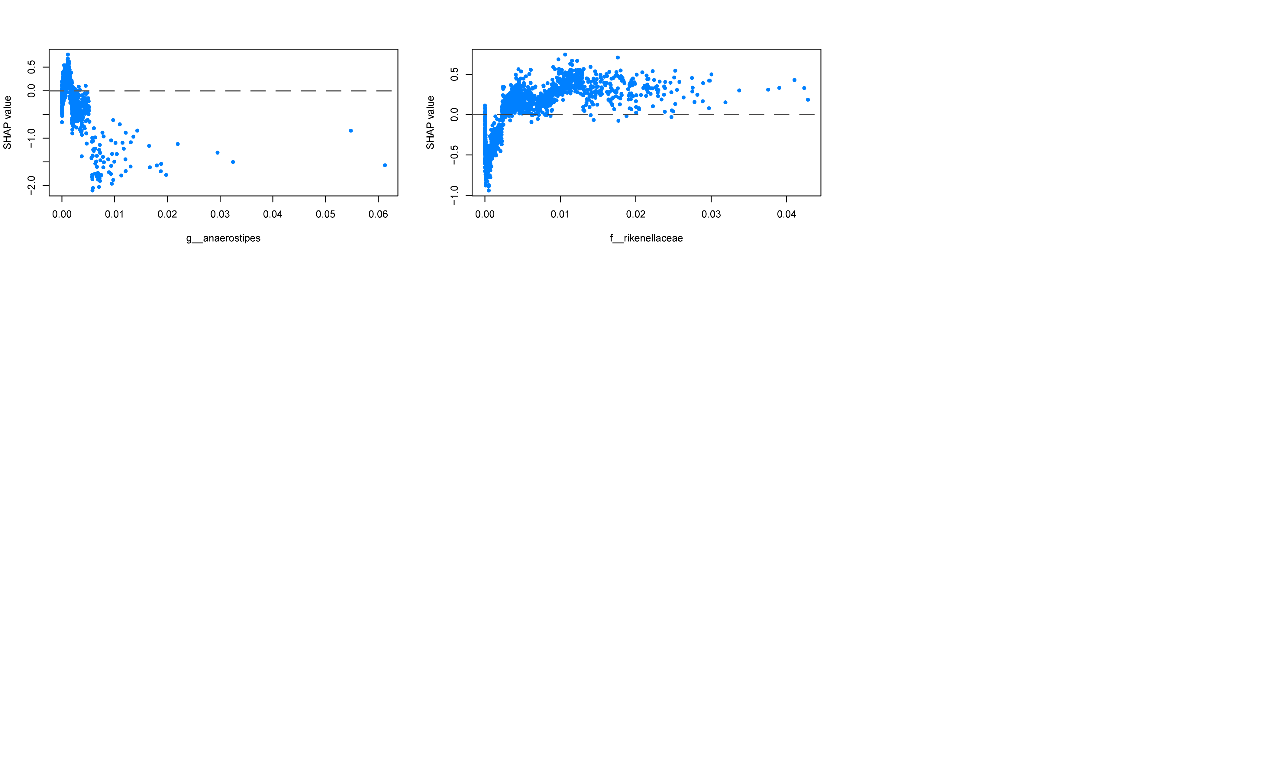


**Note:** The SHAP dependence plot shows how lightGBM prediction output is affected by a selected gut microbiome feature for each sample. The feature variable is represented by the X-axis, and the SHAP value for the feature variable is represented by the Y-axis. If the SHAP value is larger than zero, the feature may increase the risk of non-alcoholic fatty liver disease for the given sample; if not, the disease risk may decrease.
