# Supplementary material for: Gut microbiome features and metabolites in non-alcoholic fatty liver disease among community-dwelling middle-aged and older adults
Source: BMC Med. 2024 Mar 7;22:104. doi: 10.1186/s12916-024-03317-y (PMC10921631; doi:10.1186/s12916-024-03317-y)
Supplement: Supplementary file 3 — Additional file 3. Details of the association between the MRS and NAFLD. Table S1. Associations of baseline adiposity and dietary factors with microbiome risk score (MRS) in discovery cohort. Table S2. Association of the gut microbiome risk score (MRS) with NAFLD. Table S3. Association of the quartiles of gut MRS with NAFLD in the 3 cohorts. [file 12916_2024_3317_MOESM3_ESM.docx]

**Additional file 3. Details of the association between the MRS and NAFLD.**

## Table S1. Associations of baseline adiposity and dietary factors with microbiome risk score (MRS) in discovery cohort.

| **Characteristics** | **Reference** | **Beta** | **OR** | **LL** | **UL** | ***P-*value** |
| --- | --- | --- | --- | --- | --- | --- |
| Age |  | -0.007 | 0.993 | 0.971 | 1.015 | 0.515 |
| Sex |  |  |  |  |  |  |
| Male | Female | -0.649 | 0.523 | 0.382 | 0.714 | **<0.001** |
| Marital status |  |  |  |  |  |  |
| Others | Married or cohabiting | -0.255 | 0.775 | 0.546 | 1.101 | 0.154 |
| Education status |  |  |  |  |  |  |
| High school or secondary school | Junior and below | -0.133 | 0.876 | 0.645 | 1.189 | 0.394 |
| College and above | Junior and below | -0.353 | 0.702 | 0.498 | 0.991 | 0.044 |
| Income |  |  |  |  |  |  |
| 501~1500 | ≤500 | -0.229 | 0.795 | 0.283 | 2.237 | 0.664 |
| 1501~3000 | ≤500 | -0.246 | 0.782 | 0.283 | 2.163 | 0.636 |
| ≥3001 | ≤500 | -0.743 | 0.476 | 0.151 | 1.5 | 0.205 |
| BMI |  | 0.309 | 1.362 | 1.252 | 1.481 | **<0.001** |
| Waist circumference |  | 0.082 | 1.085 | 1.058 | 1.114 | **<0.001** |
| Hip circumference |  | -0.024 | 0.976 | 0.951 | 1.002 | 0.072 |
| Current tea drinking |  |  |  |  |  |  |
| Yes | No | -0.184 | 0.832 | 0.502 | 1.378 | 0.474 |
| Current alcohol drinking |  |  |  |  |  |  |
| Yes | No | -0.193 | 0.825 | 0.642 | 1.06 | 0.132 |
| Current smoking |  |  |  |  |  |  |
| Yes | No | -0.181 | 0.834 | 0.503 | 1.382 | 0.482 |
| Total energy intake |  | 0.000 | 1.000 | 1.000 | 1.000 | 0.843 |
| Vegetable intake |  | 0.000 | 1.000 | 0.999 | 1.000 | 0.424 |
| Fish intake |  | 0.001 | 1.001 | 0.997 | 1.004 | 0.745 |
| Red and processed meat intake |  | -0.001 | 0.999 | 0.997 | 1.002 | 0.474 |
| Fruit intake |  | 0.000 | 1.000 | 0.999 | 1.002 | 0.390 |
| Yogurt intake |  | 0.000 | 1.000 | 0.999 | 1.001 | 0.655 |

**Note:** Logistic regression was used to estimate the odds ratio (OR) and 95% confidence interval (CI) of NAFLD per-1 unit change in each baseline adiposity and dietary factors. Beta is correlation coefficient of baseline adiposity and dietary factors with MRS in logistic regression. The model was adjusted for sex, age, marital status, education, income, current smoking, current tea drinking, total energy intake.

**Abbreviations**: OR, odd ratio; LL, lower limit of confidence interval; UL, upper limit of confidence interval.

## Table S2. Association of the gut microbiome risk score (MRS) with NAFLD.

|  | **Discovery cohort** | | | **Internal validation cohort** | | | **Prospective validation cohort** | | |
| --- | --- | --- | --- | --- | --- | --- | --- | --- | --- |
|  | Beta | OR (95% CI) | *P* | Beta | OR (95% CI) | *P* | Beta | OR (95% CI) | *P* |
| **Model 1** | 0.615 | 1.85 (1.71, 1.99) | **<0.001** | 0.187 | 1.21 (1.07, 1.36) | **0.003** | **0.549** | **1.73 (1.56, 1.92)** | **<0.001** |
| **Model 2** | 0.615 | 1.85 (1.71, 1.99) | **<0.001** | 0.188 | 1.21 (1.07, 1.37) | **0.003** | **0.562** | **1.75 (1.58, 1.95)** | **<0.001** |
| **Model 3** | 0.622 | 1.86 (1.72, 2.02) | **<0.001** | 0.185 | 1.20 (1.06, 1.37) | **0.004** | **0.569** | **1.77 (1.58, 1.97)** | **<0.001** |
| **Model 4** | 0.715 | 2.04 (1.86, 2.25) | **<0.001** | 0.206 | 1.23 (1.07, 1.41) | **0.003** | **0.690** | **1.99 (1.75, 2.27)** | **<0.001** |

**Note:** Logistic regression was conducted to estimate the odds ratio (OR) and 95% confidence interval (CI) of NAFLD in each of the three cohorts, according to the gut microbiome risk score. In model 1, no covariates are adjusted. In model 2, the covariates were sex and age. In model 3, marital status, education, income, smoking, drinking, tea and total energy intake were adjusted based on model 2. In model 4, the BMI was further adjusted as sensitivity analysis.

**Abbreviations**: OR, odd ratio; 95%CI, 95% confidence interval.

## Table S3. Association of the quartiles of gut MRS with NAFLD in the 3 cohorts.

| **Cohorts** | **Median (MRS)** | **No. of cases / total no.** | **Model 1** | ***P* value** | **Model 2** | ***P* value** | **Model 3** | ***P* value** | **Model 4** | ***P* value** |
| --- | --- | --- | --- | --- | --- | --- | --- | --- | --- | --- |
|  |  |  | **OR (95%CI)** |  | **OR (95%CI)** |  | **OR (95%CI)** |  | **OR (95%CI)** |  |
| **Discovery cohort** | | | | | | | | | | |
| Quartile 1 | 4 | 74 / 307 | 1.00 (reference) |  | 1.00 (reference) |  | 1.00 (reference) |  | 1.00 (reference) |  |
| Quartile 2 | 5 | 110 / 291 | 1.84 (1.28, 2.64) | **0.001** | 2.02 (1.31, 3.12) | **0.002** | 1.83 (1.27, 2.63) | 0.001 | 1.93 (1.26, 2.96) | **0.003** |
| Quartile 3 | 6 | 187 / 322 | 4.19 (2.96, 5.94) | **<0.001** | 5.76 (3.77, 8.79) | **<0.001** | 4.16 (2.94, 5.90) | **<0.001** | 5.74 (3.80, 8.67) | **<0.001** |
| Quartile 4 | 8 | 496 / 626 | 11.98 (8.60, 16.70) | **<0.001** | 18.86 (12.53, 28.40) | **<0.001** | 11.93 (8.56, 16.63) | **<0.001** | 16.92 (11.37, 25.17) | **<0.001** |
| **Internal validation cohort** | | | | | | | | | | |
| Quartile 1 | 4 | 17 / 89 | 1.00 (reference) |  | 1.00 (reference) |  | 1.00 (reference) |  | 1.00 (reference) |  |
| Quartile 2 | 5 | 32 / 84 | 2.59 (1.28, 5.27) | **0.008** | 2.61 (1.21, 5.63) | **0.014** | 2.61 (1.28, 5.30) | **0.008** | 2.65 (1.24, 5.67) | **0.012** |
| Quartile 3 | 6 | 21 / 81 | 1.34 (0.63, 2.82) | 0.446 | 1.22 (0.55, 2.71) | 0.632 | 1.34 (0.64, 2.83) | 0.440 | 1.27 (0.57, 2.81) | 0.555 |
| Quartile 4 | 8 | 56 / 123 | 3.50 (1.83, 6.71) | **<0.001** | 3.44 (1.72, 6.86) | **<0.001** | 3.52 (1.83, 6.75) | **<0.001** | 3.47 (1.75, 6.90) | **<0.001** |
| **Prospective validation cohort** | | | | | | | | | | |
| Quartile 1 | 4 | 13 / 100 | 1.00 (reference) |  | 1.00 (reference) |  | 1.00 (reference) |  | 1.00 (reference) |  |
| Quartile 2 | 5 | 17 / 103 | 2.66 (1.08, 6.53) | **0.034** | 2.99 (1.19, 7.54) | **0.020** | 1.83 (1.27, 2.63) | **0.001** | 1.93 (1.26, 2.96) | **0.003** |
| Quartile 3 | 6 | 105 / 198 | 9.04 (4.21, 19.40) | **<0.001** | 10.49 (4.73, 23.27) | **<0.001** | 4.16 (2.94, 5.90) | **<0.001** | 5.74 (3.80, 8.67) | **<0.001** |
| Quartile 4 | 8 | 131 / 82 | 35.97 (16.00, 80.84) | **<0.001** | 42.64 (18.28, 99.46) | **<0.001** | 11.93 (8.56, 16.63) | **<0.001** | 16.92 (11.37, 25.17) | **<0.001** |

**Note.** Logistic regression was used to estimate the odds ratio (OR) and 95% confidence interval (CI) of NAFLD in the three cohorts, according to the gut microbiome risk score. In these comparisons, participants at low microbiome risk (Quartile 1) were treated as the reference group. In model 1, no covariates are adjusted. In model 2, the covariates were sex and age. In model 3, marital status, education, income, smoking, drinking, tea and total energy intake were adjusted based on model 2. In model 4, the BMI was further adjusted as sensitivity analysis.

**Abbreviations**: OR, odd ratio; 95%CI, 95% confidence interval.
