# Supplementary material for: Gut microbiome features and metabolites in non-alcoholic fatty liver disease among community-dwelling middle-aged and older adults
Source: BMC Med. 2024 Mar 7;22:104. doi: 10.1186/s12916-024-03317-y (PMC10921631; doi:10.1186/s12916-024-03317-y)
Supplement: Supplementary file 4 — Additional file 4. Details of the relationship between selected gut microbiota features and NAFLD. Table S1. Association between selected microbiome features and NAFLD in the 3 cohorts. Table S2. Logistics regression was used to estimate association between NAFLD and selected microbiome features at higher abundance (SHAP value higher than 0) and lower abundance (SHAP value lower than 0). Fig. S1. Logistics regression was used to estimate association between NAFLD and selected microbiome features at higher abundance (SHAP value higher than 0) and lower abundance (SHAP value lower than 0). [file 12916_2024_3317_MOESM4_ESM.docx]

**Additional file 4. Details of the relationship between selected gut microbiota features and NAFLD.**

## Table S1. Association between selected microbiome features and NAFLD in the 3 cohorts.

| **Microbiota** | **Discovery cohort** | |  | **Internal validation cohort** | |  | **Prospective validation cohort** | |
| --- | --- | --- | --- | --- | --- | --- | --- | --- |
|  | **Adjusted OR (95%CI)** | ***P*-value** |  | **Adjusted OR (95%CI)** | ***P*-value** |  | **Adjusted OR (95%CI)** | ***P*-value** |
| p__fusobacteria | 1.24 (1.10, 1.39) | **<0.001** |  | 1.00 (0.96, 1.02) | 0.821 |  | 1.25 (1.06, 1.47) | **0.010** |
| o__actinomycetales | 1.04 (0.94, 1.16) | 0.474 |  | 1.03 (0.82, 1.28) | 0.818 |  | 0.88 (0.73, 1.07) | 0.209 |
| o__turicibacterales | 1.00 (0.90, 1.11) | 0.959 |  | 1.08 (0.87, 1.34) | 0.492 |  | 1.00 (0.84, 1.19) | 0.992 |
| f__rikenellaceae | 0.82 (0.74, 0.91) | **0.001** |  | 0.84 (0.66, 1.06) | 0.143 |  | 0.77 (0.63, 0.93) | **0.006** |
| f__barnesiellaceae | 0.81 (0.73, 0.90) | **<0.001** |  | 0.79 (0.60, 1.03) | 0.084 |  | 0.66 (0.53, 0.83) | **<0.001** |
| f__veillonellaceae | 1.29 (1.15, 1.44) | **<0.001** |  | 1.04 (0.84, 1.29) | 0.714 |  | 1.11 (0.94, 1.31) | 0.238 |
| g__clostridiaceaeother | 1.05 (0.84, 1.17) | 0.397 |  | 0.98 (0.78, 1.22) | 0.829 |  | 1.21 (1.03, 1.43) | 0.021 |
| g__anaerostipes | 0.96 (0.87, 1.07) | 0.470 |  | 0.95 (0.75, 1.18) | 0.621 |  | 1.07 (0.91, 1.26) | 0.397 |
| g__klebsiella | 1.01 (0.91, 1.12) | 0.880 |  | 1.18 (0.95, 1.47) | 0.131 |  | 0.92 (0.77, 1.10) | 0.343 |
| s__bifidobacteriumother | 0.92 (0.83, 1.02) | 0.119 |  | 1.12 (0.90, 1.39) | 0.299 |  | 0.99 (0.84, 1.16) | 0.862 |
| s__adolescentis | 0.90 (0.81, 1.00) | **0.042** |  | 1.06 (0.85, 1.31) | 0.615 |  | 0.91 (0.75, 1.10) | 0.315 |
| s__acidifaciens | 0.94 (0.84, 1.05) | 0.257 |  | 0.95 (0.72, 1.24) | 0.702 |  | 1.05 (0.90, 1.22) | 0.535 |

**Note:** Logistic regression was used to estimate the odds ratio (OR) and 95% confidence interval (CI) of NAFLD per SD change in each selected microbiome feature. The model was adjusted for sex, age, marital status, education, income, current smoking, current tea drinking, total energy intake.

**Abbreviations**: OR, odd ratio; LL, lower limit of confidence interval; UL, upper limit of confidence interval.

## Table S2. Logistics regression was used to estimate association between NAFLD and selected microbiome features at higher abundance (SHAP value higher than 0) and lower abundance (SHAP value lower than 0).

| **Microbiota** | **Discovery cohort** | |  | **Internal validation cohort** | |  | **Prospective validation cohort** | |
| --- | --- | --- | --- | --- | --- | --- | --- | --- |
|  | **Adjusted OR (95%CI)** | ***P*-value** |  | **Adjusted OR (95%CI)** | ***P*-value** |  | **Adjusted OR (95%CI)** | ***P*-value** |
| p__fusobacteria | 1.82 (1.43, 2.32) | **<0.001** |  | 0.89 (0.53, 1.48) | 0.644 |  | 1.76 (1.27, 2.43) | **0.001** |
| o__actinomycetales | 1.51 (1.22, 1.88) | **<0.001** |  | 1.09 (0.69, 1.72) | 0.715 |  | 1.34 (0.98, 1.82) | 0.066 |
| o__turicibacterales | 0.79 (0.64, 0.98) | **0.023** |  | 0.72 (0.46, 1.12) | 0.139 |  | 0.68 (0.50, 0.93) | **0.015** |
| f__rikenellaceae | 0.85 (0.77, 1.17) | 0.618 |  | 0.99 (0.63, 1.55) | 0.955 |  | 0.98 (0.69, 1.39) | 0.914 |
| f__barnesiellaceae | 0.51 (0.41, 0.62) | **<0.001** |  | 0.82 (0.53, 1.28) | 0.380 |  | 0.56 (0.41, 0.76) | **<0.001** |
| f__veillonellaceae | 2.85 (2.26, 3.59) | **<0.001** |  | 1.49 (0.93, 2.40) | 0.098 |  | 1.71 (1.25, 2.34) | **0.001** |
| g__clostridiaceaeother | 1.64 (1.33, 2.02) | **<0.001** |  | 1.71 (1.07, 2.75) | **0.026** |  | 1.70 (1.23, 2.35) | **0.001** |
| g__anaerostipes | 0.45 (0.36, 0.56) | **<0.001** |  | 0.80 (0.49, 1.30) | 0.359 |  | 0.39 (0.28, 0.53) | **<0.001** |
| g__klebsiella | 0.48 (0.39, 0.60) | **<0.001** |  | 0.65 (0.42, 1.02) | 0.063 |  | 0.54 (0.39, 0.74) | **<0.001** |
| s__bifidobacteriumother | 0.58 (0.47, 0.71) | **<0.001** |  | 1.19 (0.76, 1.85) | 0.454 |  | 0.53 (0.38, 0.75) | **<0.001** |
| s__adolescentis | 0.42 (0.34, 0.53) | **<0.001** |  | 0.63 (0.35, 1.13) | 0.122 |  | 0.38 (0.27, 0.52) | **<0.001** |
| s__acidifaciens | 0.73 (0.59, 0.90) | **0.003** |  | 0.76 (0.48, 1.21) | 0.247 |  | 0.59 (0.42, 0.82) | **0.002** |

**Note:** The model was adjusted for sex, age, marital status, education, income, current smoking, current tea drinking, total energy intake.

**Abbreviations**: OR, odd ratio; LL, lower limit of confidence interval; UL, upper limit of confidence interval.

## Fig. S1. Logistics regression was used to estimate association between NAFLD and selected microbiome features at higher abundance (SHAP value higher than 0) and lower abundance (SHAP value lower than 0).

**
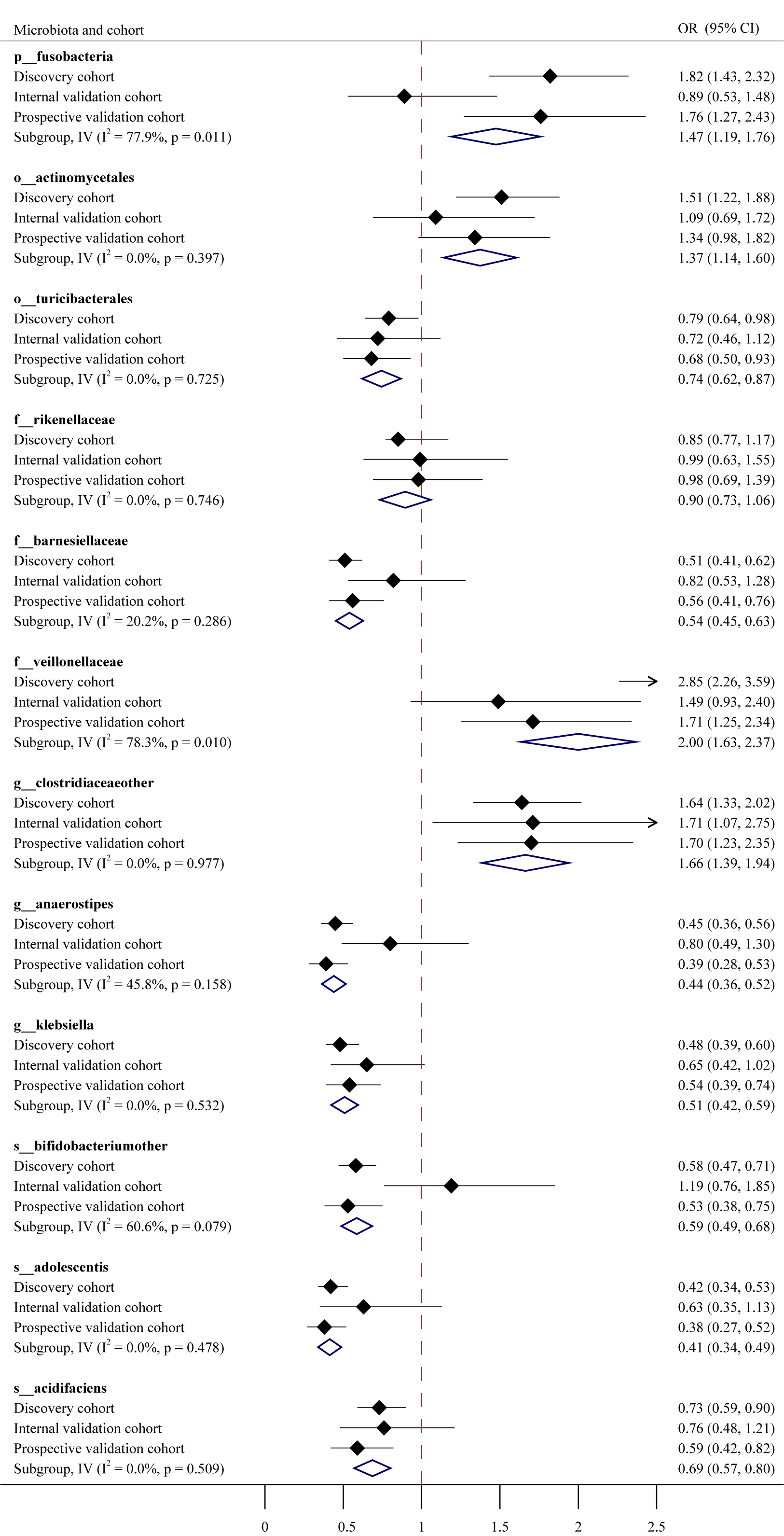
**

**Note:** The model was adjusted for sex, age, marital status, education, income, current smoking, current tea drinking, total energy intake.

**Abbreviations**: OR, odd ratio; LL, lower limit of confidence interval; UL, upper limit of confidence interval.
