# Supplementary material for: Gut microbiome features and metabolites in non-alcoholic fatty liver disease among community-dwelling middle-aged and older adults
Source: BMC Med. 2024 Mar 7;22:104. doi: 10.1186/s12916-024-03317-y (PMC10921631; doi:10.1186/s12916-024-03317-y)
Supplement: Supplementary file 5 — Additional file 5. Details of identification of significantly different metabolites and the association between SDM with gut microbiota features. Fig. S1. The Principal Component Analysis (PCA) score plot of the first two principal components for metabolite levels among groups by MRS in the discovery cohort. Fig. S2. Univariate analysis results of differential metabolites in different groups by MRS in the discovery cohort. Fig. S3. Important metabolites selected by volcano plot in different groups by MRS in the discovery cohort. Fig. S4. The Principal Component Analysis (PCA) score plot of the first two principal components for metabolite levels among groups by whether with NAFLD or not in the discovery cohort. Fig. S5. Univariate analysis results of differential metabolites in different groups by whether with NAFLD or not in the discovery cohort. Fig. S6. Important metabolites selected by volcano plot in different groups by whether with NAFLD or not in the discovery cohort. Table S1. Four significant different metabolites (SDMs) in faecal samples among discovery cohort (higher MRS group vs. lower MRS group). Table S2. One significant different metabolites (SDMs) in faecal samples among discovery cohort (NAFLD group vs. control group). Table S3. Correlations between selected microbiomes and Taurocholic acid. [file 12916_2024_3317_MOESM5_ESM.docx]

**Additional file 5. Details of identification of significantly different metabolites and the association between SDM with gut microbiota features.**

**Fig. S1.** The Principal Component Analysis (PCA) score plot of the first two principal components for metabolite levels among groups by MRS in the discovery cohort.


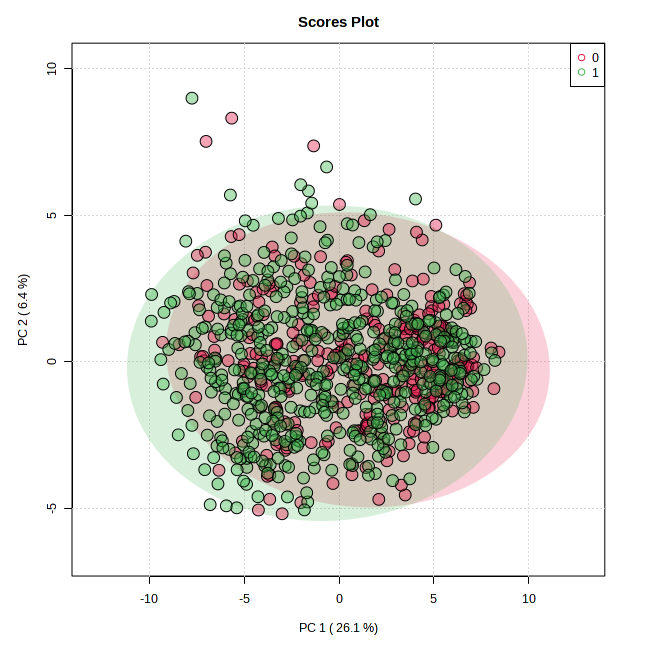


**Note**: The samples in the score plots were classified by MRS, with red (legend 0) denoting the lower MRS group and green (legend 1) denoting higher MRS group.

**Fig. S2.** Univariate analysis results of differential metabolites in different groups by MRS in the discovery cohort.
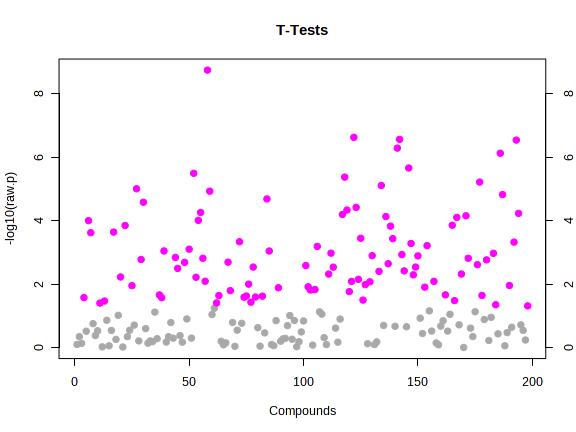


**Note**: Symbol in violet denotes a statistically significant Benjamini-Hochberg adjusted false discovery rate (FDR) of the Student’s t-test (FDR<0.05) whereas symbol in grey indicates no significant FDR.

**Fig. S3.** Important metabolites selected by volcano plot in different groups by MRS in the discovery cohort.


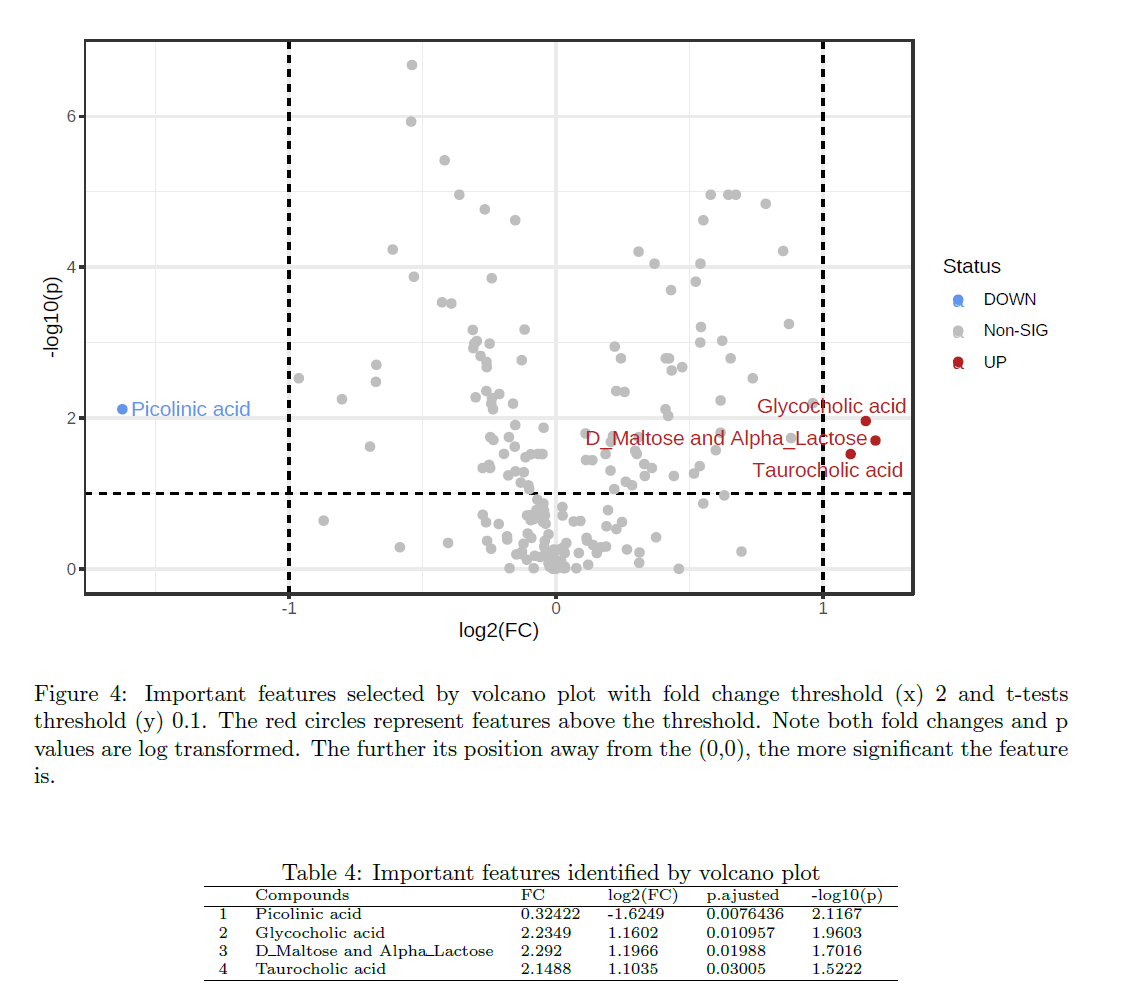


**Note**: Red indicates the upregulated metabolites expression level of the NAFLD patient group, green indicates the downregulated metabolites expression level of the NAFLD patient group, and black indicated insignificant differential expression; both FC and P-value are log transformed; the further its position away from the (0, 0), the more significant the feature is.

**Fig. S4.** The Principal Component Analysis (PCA) score plot of the first two principal components for metabolite levels among groups by whether with NAFLD or not in the discovery cohort.


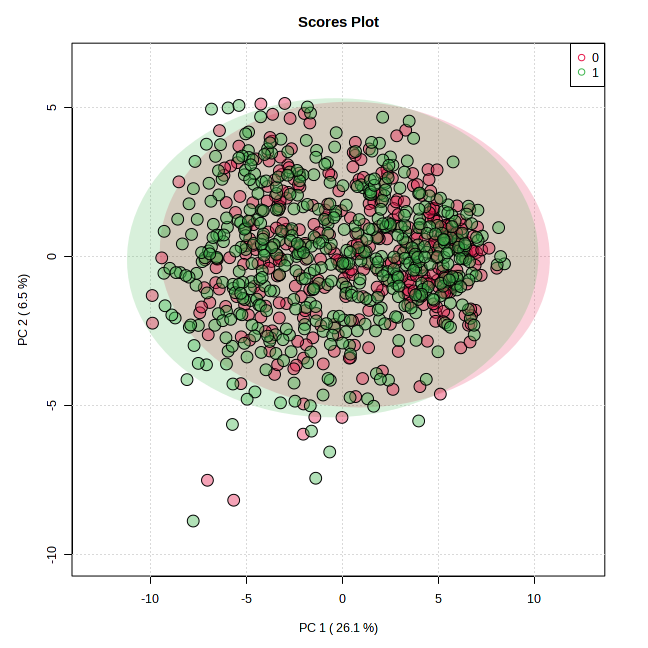


**Note**: The samples in the score plots were classified by MRS, with red (legend 0) denoting the lower MRS group and green (legend 1) denoting higher MRS group.

**Fig. S5.** Univariate analysis results of differential metabolites in different groups by whether with NAFLD or not in the discovery cohort.
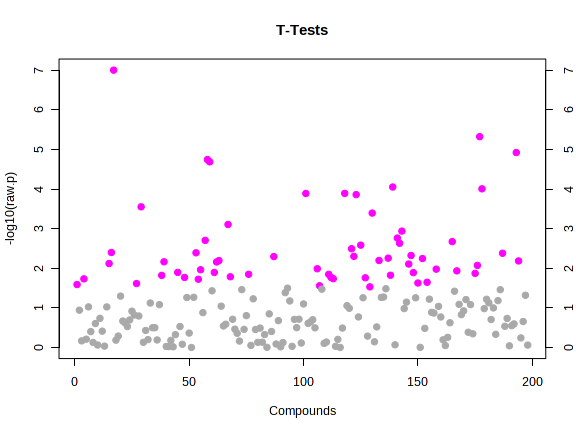


**Note**: Symbol in violet denotes a statistically significant Benjamini-Hochberg adjusted false discovery rate (FDR) of the Student’s t-test (FDR<0.05) whereas symbol in grey indicates no significant FDR.

**Fig. S6.** Important metabolites selected by volcano plot in different groups by whether with NAFLD or not in the discovery cohort.

**
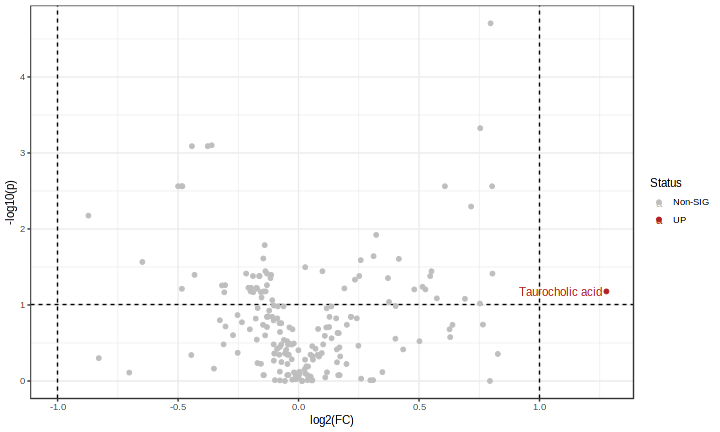
**

**Note**: Red indicates the upregulated metabolites expression level of the NAFLD patient group, green indicates the downregulated metabolites expression level of the NAFLD patients group, and black indicated insignificant differential expression; both FC and P-value are log transformed; the further its position away from the (0, 0), the more significant the feature is.

**Table S1.** Four significant different metabolites (SDMs) in faecal samples among discovery cohort (higher MRS group vs. lower MRS group).

|  | **Volcano** | | | |  | **T-test** | | | |
| --- | --- | --- | --- | --- | --- | --- | --- | --- | --- |
|  | FC | log2(FC) | FDR | -Log10(p) |  | t | *P* | -Log10(p) | FDR |
| Glycocholic acid | 2.235 | 1.160 | 0.003 | 2.543 |  | 3.483 | 0.001 | 3.283 | 0.003 |
| D_Maltose and Alpha_Lactose | 2.292 | 1.197 | 0.004 | 2.363 |  | 3.332 | 0.001 | 3.047 | 0.004 |
| Taurocholic acid | 2.149 | 1.104 | 0.028 | 1.560 |  | 2.571 | 0.010 | 1.987 | 0.028 |
| Picolinic acid | 0.324 | -1.625 | 0.056 | 1.248 |  | -2.222 | 0.027 | 1.576 | 0.056 |

**Note:** The FC describes the ratio of expression in metabolites between NAFLD patients and control group.

**Abbreviations**: FC, fold change, FDR, false discovery rate.

**Table S2.** One significant different metabolites (SDMs) in faecal samples among discovery cohort (NAFLD group vs. control group).

|  | **Volcano** | | | |  | **T-test** | | | |
| --- | --- | --- | --- | --- | --- | --- | --- | --- | --- |
|  | FC | log2(FC) | FDR | -Log10(p) |  | t | *P* | -Log10(p) | FDR |
| Taurocholic acid | 2.424 | 1.278 | 0.066 | 1.178 |  | 2.382 | 0.017 | 1.759 | 0.066 |

**Note:** The FC describes the ratio of expression in metabolites between NAFLD patients and control group.

**Abbreviations**: FC, fold change, FDR, false discovery rate.

**Table S3.** Correlations between selected microbiomes and Taurocholic acid.

| **Metabolites** | **Microbiomes** | **Correlation coefficients** | ***P*-value** |
| --- | --- | --- | --- |
| Taurocholic acid | p__fusobacteria | 0.198 | **<0.001** |
|  | o__actinomycetales | -0.142 | **<0.001** |
|  | o__turicibacterales | -0.070 | **0.041** |
|  | f__barnesiellaceae | -0.357 | **<0.001** |
|  | f__rikenellaceae | -0.345 | **<0.001** |
|  | f__veillonellaceae | 0.064 | 0.061 |
|  | g__anaerostipes | 0.010 | 0.778 |
|  | g__clostridiaceaeother | 0.099 | **0.004** |
|  | g__klebsiella | -0.038 | 0.274 |
|  | s__acidifaciens | 0.004 | 0.911 |
|  | s__adolescentis | -0.085 | **0.014** |
|  | s__bifidobacteriumother | -0.036 | 0.292 |

**Note**: The Spearman correlation coefficients between 12 microbiomes selected through lightGBM and the host faecal metabolites were calculated in the discovery cohort.
